# Unveiling Structural Heterogeneity and Evolutionary Adaptations of Heteromultimeric Bacterioferritin Nanocages

**DOI:** 10.1002/advs.202409957

**Published:** 2025-04-01

**Authors:** Yingxi Li, Weiwei Wang, Wei Wang, Xing Zhang, Jinghua Chen, Haichun Gao

**Affiliations:** ^1^ State Key Laboratory for Vegetation Structure Function and Construction (VegLab) Institute of Microbiology and College of Life Sciences Zhejiang University Hangzhou 310058 China; ^2^ Center of Cryo Electron Microscopy Zhejiang University School of Medicine Hangzhou 310058 China

**Keywords:** bacterioferritin, ferritin evolution, iron storage, nanocage, structural heterogeneity

## Abstract

Iron‐storage bacterioferritins (Bfrs), existing in either homo‐ or hetero‐multimeric form, play a crucial role in iron homeostasis. While the structure and function of homo‐multimeric bacterioferritins (homo‐Bfrs) have been extensively studied, little is known about the assembly, distinctive characteristics, or evolutionary adaptations of hetero‐multimeric bacterioferritins (hetero‐Bfrs). Here, the cryo‐EM structure and functional characterization of a bacterial hetero‐Bfr (*So*Bfr12) are reported. Compared to homo‐Bfrs, although *So*Bfr12 exhibits a conserved spherical cage‐like dodecahedron, its pores through which ions traverse exhibit substantially increased diversity. Importantly, the heterogeneity has significant impacts on sites for ion entry, iron oxidation, and reduction. Moreover, evolutionary analyses reveal that hetero‐Bfrs may represent a new class within the Bfr subfamily, consisting of two different types that may have evolved from homo‐Bfr through tandem duplication and directly from ferritin (Ftn) via dispersed duplication, respectively. These results reveal remarkable structural and functional features of a hetero‐Bfr, enabling the rational design of nanocages for enhanced iron‐storing efficiency and for other specific purposes, such as drug delivery vehicles and nanozymes.

## Graphical abstract

This study explores the structural heterogeneity and evolutionary adaptations of heteromultimeric bacterioferritins. Hetero‐Bfrs, evolved from either a homo‐Bfr or Ftn by gene duplications, differ from homo‐Bfrs substantially in the mechanisms underpinning iron oxidation and reduction despite high similarity in overall structure. The findings provide insights into how hetero‐Bfrs evolve to be more specialized for storing iron.

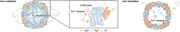

## 1 Introduction

Iron is an essential nutrient for almost all living organisms and is involved in various biological processes, such as DNA synthesis, respiration, photosynthesis, and oxidative stress response.^[^ ^1^ ^]^ Microorganisms have evolved many exquisite mechanisms to uptake iron from their surroundings, frequently to levels over the physiological demands. However, an excess of intracellular free iron tends to induce the formation of free radicals, primarily reactive oxygen species (ROS), which are toxic to cells.^[^ ^2^ ^]^ Conceivably, to maintain iron homeostasis in the cell, microorganisms have also evolved mechanisms for sequestering/storing excessive iron.^[^ ^3^, ^4^, ^5^ ^]^ The sequestered iron can be released and utilized by the cell when required.^[^ ^6^, ^7^, ^8^ ^]^

In prokaryotes, iron‐storage nanocages formed by proteins of the ferritin family serve as the primary depot site for iron sequestration or storage.^[^ ^9^, ^10^, ^11^ ^]^ In addition to their physiological functions, ferritin‐like proteins are considered an ideal platform for precisely manufacturing diverse nanostructures, which have been widely used in developing nanozymes and drug delivery systems.^[^ ^12^, ^13^ ^]^ In recent years, ferritin‐based drug delivery systems, called either ferritin drug carriers or ferritin‐drug conjugates, have emerged as a promising class of therapeutics in targeted cancer therapy.^[^ ^12^, ^13^ ^]^ There are three major subfamilies in the ferritin family: the typical Ftn, Bfr, and DNA‐binding protein from starved cell (Dps) according to their structural and functional differences.^[^ ^9^, ^10^, ^11^ ^]^ In spite of low sequence identities, structures of the subunits of these three subfamilies are highly conserved, with each subunit consisting of a four‐helix bundle and a short C‐terminal helix.^[^ ^14^ ^]^ In both Ftn and Bfr, 24 subunits assemble into a nearly spherical shell of 12 nm in diameter that can store ≈2500 iron atoms, whereas the nanocage formed by Dps, which is 9 nm in diameter and can only hold up to ≈500 iron atoms, is in a distinct dodecameric form.^[^ ^15^ ^]^ While robust iron‐storage capacity is common to all three subfamilies, Bfrs function primarily in regulating iron homeostasis, whereas Ftn and Dps participate in more diverse biological processes, such as sensing stress and/or combating oxidative stress.^[^ ^3^ ^]^

Unlike single‐subunit bacterial Ftn, Bfr can exist as a homo‐ or hetero‐complex, which assembles from two distinct subunits in a 1:1 ratio.^[^ ^16^, ^17^ ^]^ Homo‐Bfrs, such as those from *Pseudomonas aeruginosa* and *Escherichia coli*, which have been extensively studied, are structurally a 4‐3‐2‐fold symmetric nanocage composed of 12 identical homodimers with 12 heme molecules bound between two subunits at a twofold symmetric binding site (Figure S1, Supporting Information).^[^ ^18^, ^19^ ^]^ As a spherical structure, the homo‐Bfr nanocage has six 4‐fold pores, eight 3‐fold pores, and twenty‐four B‐pores, which serve as the entry and exit channels of various ions, including Fe^2+^, Fe^3+^, and phosphate.^[^ ^20^, ^21^, ^22^ ^]^ The B‐pores are formed at the asymmetric intersecting site of three subunits and lined with negatively charged residues, while the 4‐fold pores are lined with highly conserved residues (‐NYLQ‐), which bind a monovalent or divalent cation other than iron in most crystal structures of Bfrs,^[^ ^3^, ^22^ ^]^ and the 3‐fold pores, along the 3‐fold symmetry axes, are lined by alternating layers of negatively and positively charged residues.^[^ ^23^ ^]^ The interior of the four‐helix bundle contains a ferroxidase center (FC), which catalyzes oxidation of Fe^2+^ to Fe^3+^. To store free iron, homo‐Bfr takes Fe^2+^ through B‐pores, or arguably in some cases through 4‐fold pores, into the FC, where the conversion of Fe^2+^ to Fe^3+^ occurs using oxidants such as O_2_ or H_2_O_2_.^[^ ^24^, ^25^ ^]^ Subsequently, Fe^3+^ is initially mineralized in close proximity to the 4‐fold pores, and Fe^3+^‐mineral gradually extends into the central cavity of the Bfr nanocage over time.^[^ ^26^, ^27^ ^]^ When needed, heme molecules transfer electrons from Bfr‐associated ferredoxins (Bfd) to the Fe^3+^‐mineral core for iron reduction, allowing the release of stored iron.^[^ ^19^, ^23^, ^28^, ^29^ ^]^

Even though hetero‐Bfrs are widespread in bacteria, they are underexplored. Only recently, it was demonstrated that hetero‐Bfr from *Magnetospirillum gryphiswaldense* MSR‐1 is composed of two distinct subunits; the heme‐free Bfr1 possesses ferroxidase activity while the heme‐binding Bfr2 is attributed to iron reduction.^[^ ^16^ ^]^ Hence, the functioning mechanism of the hetero‐Bfr nanocages at the molecular level remains largely unknown. In this study, we attempted to address how this type of Bfrs assemble into heteromultimers and how they differ from their homo counterparts in efficacy and in evolution, with a hetero‐Bfr from *Shewanella oneidensis*. *S. oneidensis* is a facultative γ‐proteobacterium of high iron demand because it utilizes a large repertoire of iron‐containing proteins for its respiratory diversity.^[^ ^30^, ^31^ ^]^ By solving a 2.6 Å‐resolution cryo‐EM structure of *So*Bfr12, we found that although *So*Bfr12 adopts a conserved spherical cage‐like dodecahedron, its pores for ion entry, ferroxidase centers, and heme binding and iron reduction sites are significantly different from those of homo‐Bfrs. Subsequent biochemical and physiological characterizations substantiated that the heme‐free and heme‐binding dimers of hetero‐Bfrs develop specificity for iron oxidation and reduction, respectively. Furthermore, evolutionary analyses revealed a novel perspective on the evolutionary pattern for hetero‐Bfrs, and surprisingly, subunits of homo‐ and hetero‐Bfrs, when expressed all together in vivo, could assemble indistinguishably into a functional nanocage.

## 2 Results

### 2.1 Gene organizations for Hetero‐Bfrs and Functional Characterization of *So*Bfr12

**Figure 1:**
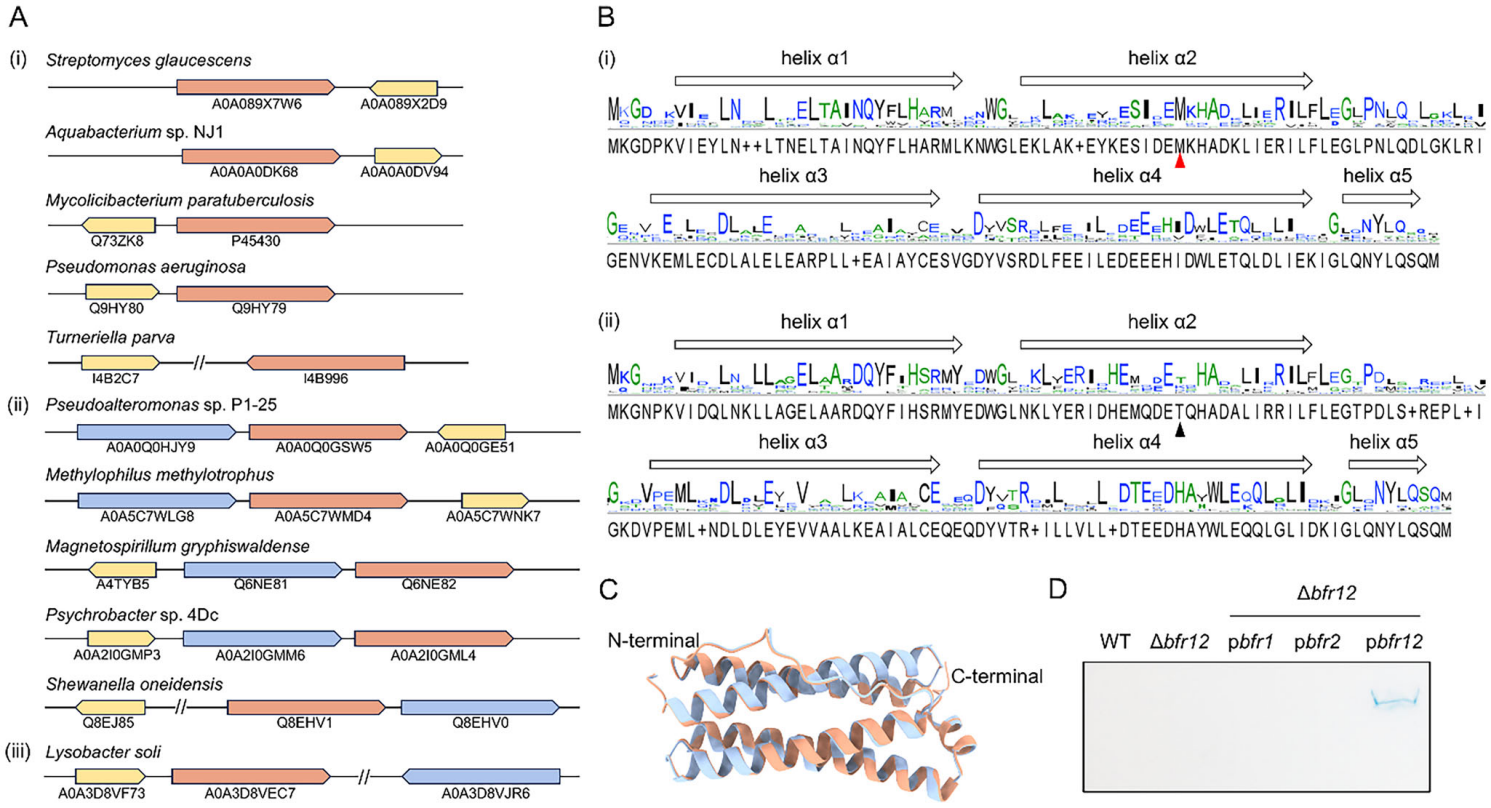
Gene organization and characterization of hetero‐Bfrs. A) The arrangement of *bfr* and *bfd* genes in bacteria. (i–iii) is the arrangement of *bfr* genes of homo‐, TD‐type hetero‐, and DSD‐type hetero‐Bfrs. The *bfr* genes encoding heme‐binding and heme‐free subunits are coloured in salmon and steel blue, respectively, and *bfd* genes are coloured in yellow. Genes preceding and following the symbol // are not next to each other on the chromosome. B) The sequence alignment of heme‐free and heme‐binding subunits. The conserved residue Met^52^, essential for heme binding, is marked by a red triangle. Residue 52 in heme‐free subunits is marked by a black triangle. C) The structural alignment of the *So*Bfr1 (AFDB: AF‐Q8EHV0‐F1) and *So*Bfr2 (AFDB: AF‐Q8EHV1‐F1). The structures of *So*Bfr1 and *So*Bfr2 are colored in steel blue and salmon, respectively. D) The iron staining of strains WT, Δ*bfr12*, Δ*bfr12*/p*bfr1*, Δ*bfr12*/p*bfr2*, and Δ*bfr12*/p*bfr12* by Native‐PAGE. The expression of a copy of an indicated gene was driven by the IPTG‐inducible promoter of Ptac within pHGEN‐Ptac. Cells at the mid‐exponential phase were collected for iron staining, either without induction or after being induced with 0.5 mmol IPTG for 2 h.

Although multiple proteins with at least partial homology to proteins belonging to the ferritin family are often present in prokaryotes, it is well established that hetero‐Bfrs are composed of two distinct subunits only.^[^ ^16^, ^32^ ^]^ These two *bfr* genes in the context of genomes can be organized into a single operon, which is derived from tandem duplication (TD) as in *S. oneidensis* and *M. gryphiswaldense*, or scattered on the chromosome, a result of dispersed duplication (DSD), as in *Lysobacter soli* (**Figure** **1**A). Interestingly, the order of the TD‐type *bfr* genes, which are apparently a result of gene duplication from a *bfr* gene for homo‐Bfr, could be opposite; in some microorganisms (i.e., *M. gryphiswaldense*), the gene for the heme‐free subunit is up front, whereas in some others (i.e., *S. oneidensis*) it follows. The occurrence of these two patterns appears to be comparable (i.e., “heme‐free/heme‐binding” to “heme‐binding/heme‐free” 94:112 in 206 genomes hosting a TD‐type hetero‐Bfr), implying that after gene duplication, either descendant gene can evolve to encode a heme‐free subunit (Figure S2A, Supporting Information). Additionally, we found that, in line with function association, the *bfd* gene is often found in proximity of the *bfr* operon (in the case of DSD‐type, one of two *bfr* operons). However, the orientation of the *bfd* gene is rather flexible relative to the *bfr* operon, either head‐on or co‐oriented in terms of transcription (Figure 1A). A similar scenario is observed in bacteria hosting a homo‐Bfr, as in *P. aeruginosa* or in *Turneriella parva*. It is worth mentioning that the *bfr* and *bfd* genes have never been found to be co‐transcribed. Thus, the *bfr* genes, along with the *bfd* gene, barely impact genome stability through transcription–replication conflicts during evolution.^[^ ^33^ ^]^

Sequence identity between the heme‐free and heme‐binding subunits of any bacterial hetero‐Bfr is not higher than 45%, implying that they have undergone a long history of mutation accumulation (Figure S2B, Supporting Information). Despite this, these two subunits share a highly similar secondary structure featured by 5 α‐helices and apparently have a similar set of conserved residues (Figure 1B), suggesting an overall conservation in functionality. Among the differences in amino acid residues that may have altered the functional properties, in particular, Met^52^ within heme‐free subunits is replaced by other residues, resulting in the loss of heme‐binding capacity (Figure 1B). In addition, the 3D structures of these two subunits available in AlphaFold DB are nearly identical;^[^ ^34^, ^35^ ^]^ for example, *So*Bfr1 and *So*Bfr2 have confidence scores (pLDDT) of 98 for entire proteins (Figure 1C).

We have previously shown that among ferritin‐like proteins, *So*Bfr12 complex is the only one in the wild‐type (WT) strain of *S. oneidensis* that can be detected on Native‐PAGE by iron staining assay when grown in iron‐rich LB broth (supplemented with 1 mmol FeSO_4_), and this Prussian blue band becomes invisible from the WT cells in the absence of induction by excess iron.^[^ ^36^ ^]^ In preparation of *So*Bfr12 for structure determination, we first substantiated that both subunits are required for iron‐storing activity with WT, a strain devoid of the *bfr* operon (Δ*bfr12*) and its genetically complemented strains with *bfr1*, *bfr2*, or both. Consistently, the iron staining assay failed to detect *So*Bfr12 from WT cells grown in LB broth, and the same results were obtained with Δ*bfr12* expressing either of the *bfr* genes (Figure 1D). Only when both *bfr1* and *bfr2* are forcibly co‐overexpressed, a Prussian blue band was observed, indicating that both subunits are essential to iron‐storing activity and heme‐binding subunits, unlike those of homo‐Bfrs, on their own cannot independently assemble a functional complex (Figure 1D).

### 2.2 Overall Structure of *So*Bfr12

Recombinant *So*Bfr12 was overexpressed in *E. coli* from a DNA fragment encoding *So*Bfr2 and *So*Bfr1 with a Strep‐tag II within pET28a(+). After column affinity chromatography and size exclusion chromatography, proteins were purified and migrated as a complete 24‐mer complex composed of both subunits, which appear in similar quantity (Figure S3A,B, Supporting Information). Highly purified portions of *So*Bfr12 were pooled for subsequent biochemical analysis and structure determination with cryo‐EM. A total of 6940 images were collected and processed with CryoSPARC 4.4.^[^ ^37^ ^]^ After rounds of 2D and 3D classifications, 736353 particles were used for 3D refinement with a *C*1 symmetry, which yielded a final reconstruction with a resolution of 2.62 Å (Figure S4 and Table S1, Supporting Information).

**Figure 2:**
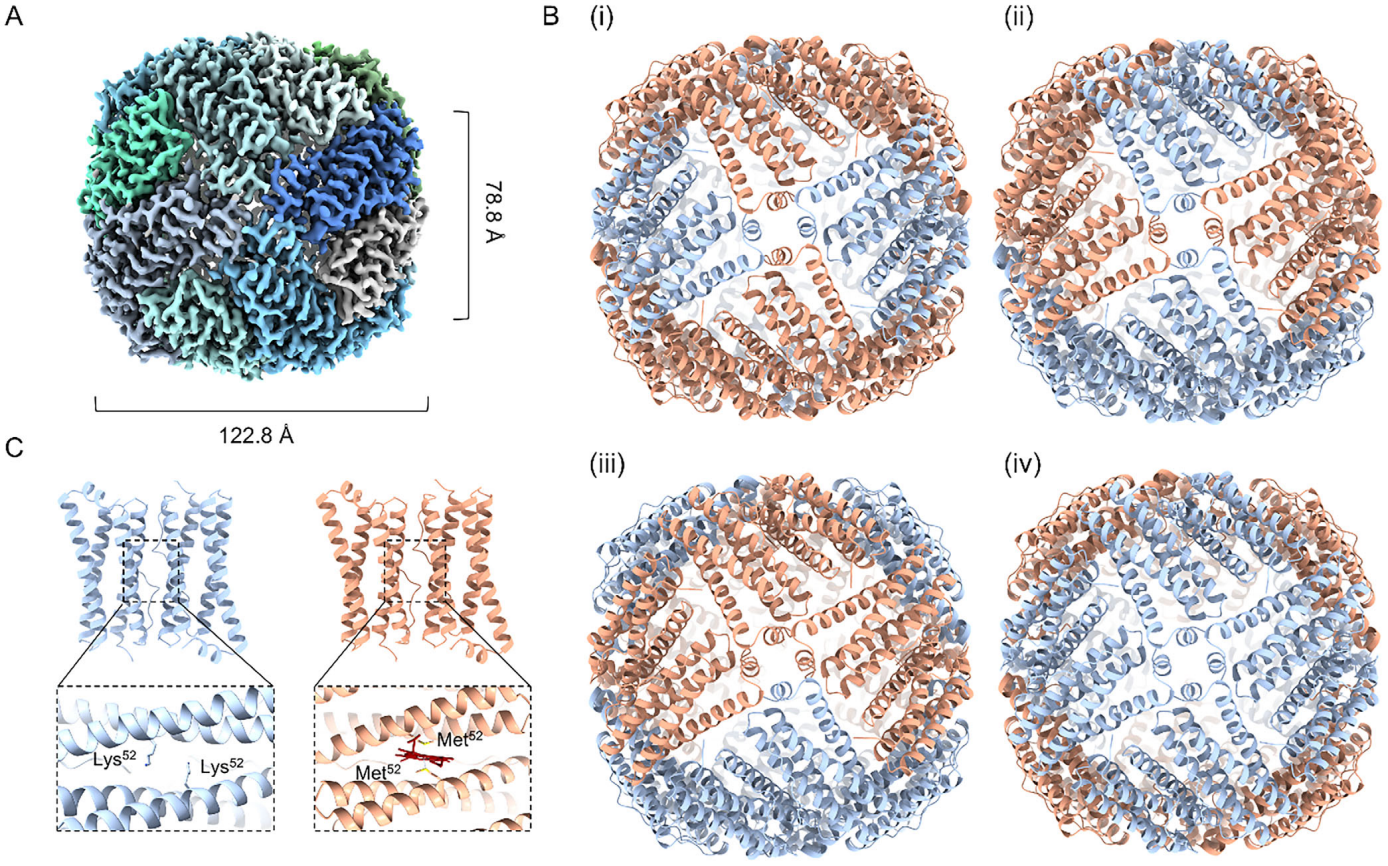
Overall structure of the *So*Bfr12 nanocage. A) Cryo‐EM density map of *So*Bfr12 at a resolution of 2.62 Å. B) Cartoon model of *So*Bfr12 from four different angles (i–iv). The overall structure of *So*Bfr12 consists of six *So*Bfr2 dimers (salmon) and six *So*Bfr1 dimers (steel blue) in a *C*2 symmetry defined by the two‐fold axis connecting one subtype III 4‐fold pore (i) and one subtype I 4‐fold pore (iv). C) *So*Bfr1 (left, steel blue) and *So*Bfr2 dimers (right, salmon). Shown are close‐up views of lysine residues (Lys^52^) and conserved heme‐binding methionine residues (Met^52^) at the interface of *So*Bfr1 and *So*Bfr2 dimers.

The overall structure of *So*Bfr12 exhibits a highly conserved nanocage architecture with the external and internal diameters of 122.8 and 78.8 Å, respectively (**Figure** **2**A). Differing from homo‐Bfrs, in which the subunits are arranged with *O* symmetry,^[^ ^18^, ^38^, ^39^ ^]^ the *So*Bfr12 near‐spherical nanocage comprises 12 *So*Bfr1 and 12 *So*Bfr2 subunits that are arranged in a *C*2 symmetry (Figure 2B). During assembly, 12 subunits of the same type first form 6 homodimers (Figure 2B), in which two subunits are arranged in an antiparallel direction, and no heterodimers were observed (Figure 2C). In accord with the prediction by AlphaFold, the structures of *So*Bfr1 and *So*Bfr2 are almost identical, containing a four‐helix bundle and a short C‐terminal helix, a common characteristic of Bfr subfamily members (Figure 2C). Six heme‐free homodimers and six heme‐binding homodimers then assemble into a hetero‐Bfr nanocage in a fixed pattern. The most significant difference between *So*Bfr12 and homo‐Bfrs lies in that there is no heme bound at the interface of two *So*Bfr1 subunits because of the lack of the conserved heme binding residue (Met^52^), and as a result, *So*Bfr1 and *So*Bfr2 are unlikely to function equivalently.

### 2.3 Pores of *So*Bfr12

**Figure 3:**
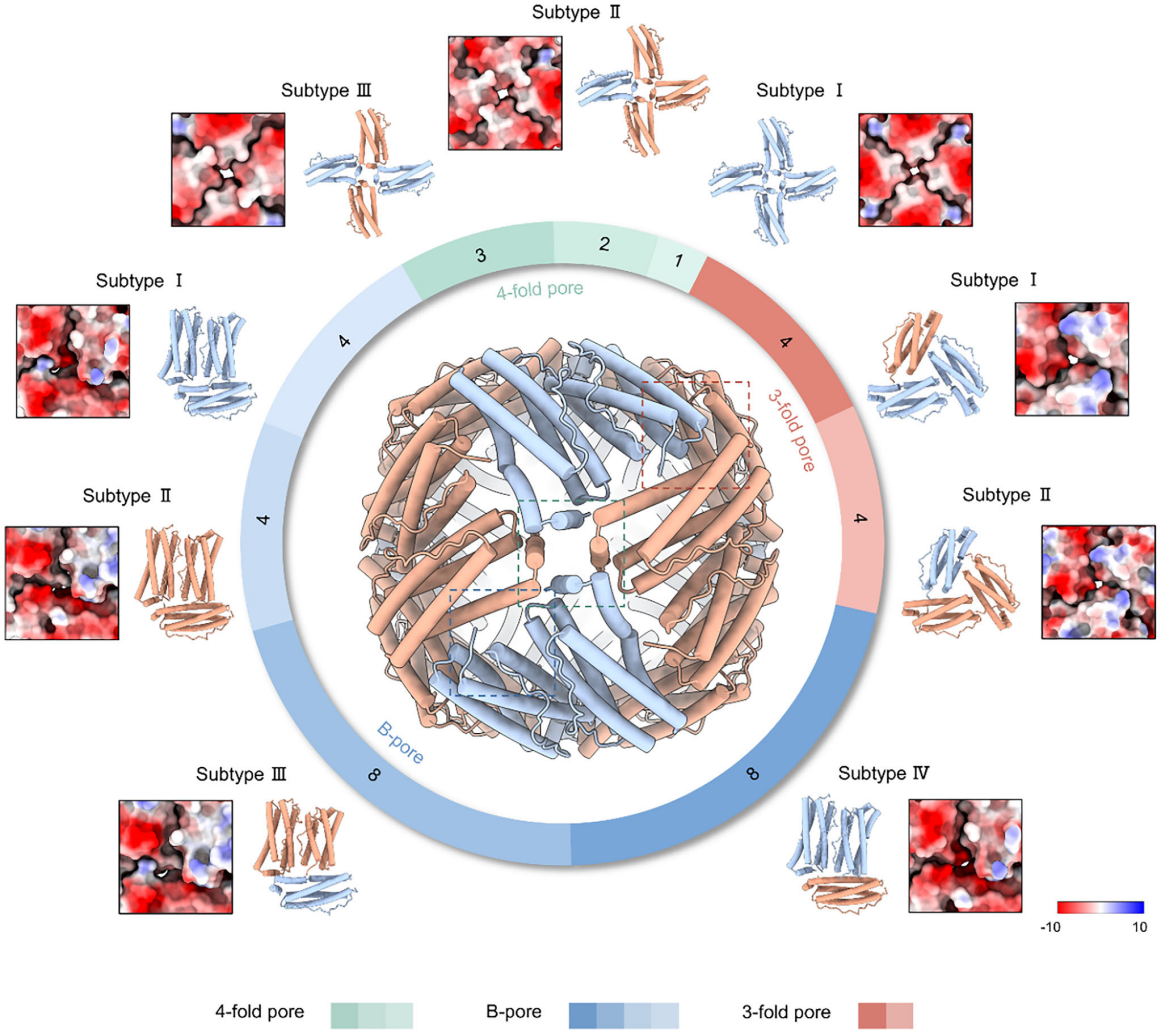
Composition and surface electrostatic potential of B‐pores, 4‐fold pores, and 3‐fold pores in *So*Bfr12. The subtypes of 4‐fold pores, B‐pores, and 3‐fold pores composed of *So*Bfr1 (steel blue) and *So*Bfr2 (salmon) subunits in different quantitative ratios are displayed on a pie chart in proportion to their quantity. The schemes in green, blue, and red colours represent 4‐fold pores, B‐pores, and 3‐fold pores, respectively. The locations of these pores in the nanocage are shown in the square boxes. The total number of the pores for each subtype is given on the pie ring. The surface electrostatic potential for each subtype of pores, calculated by ChimeraX according to Coulomb's law, is also shown.

**Figure 4:**
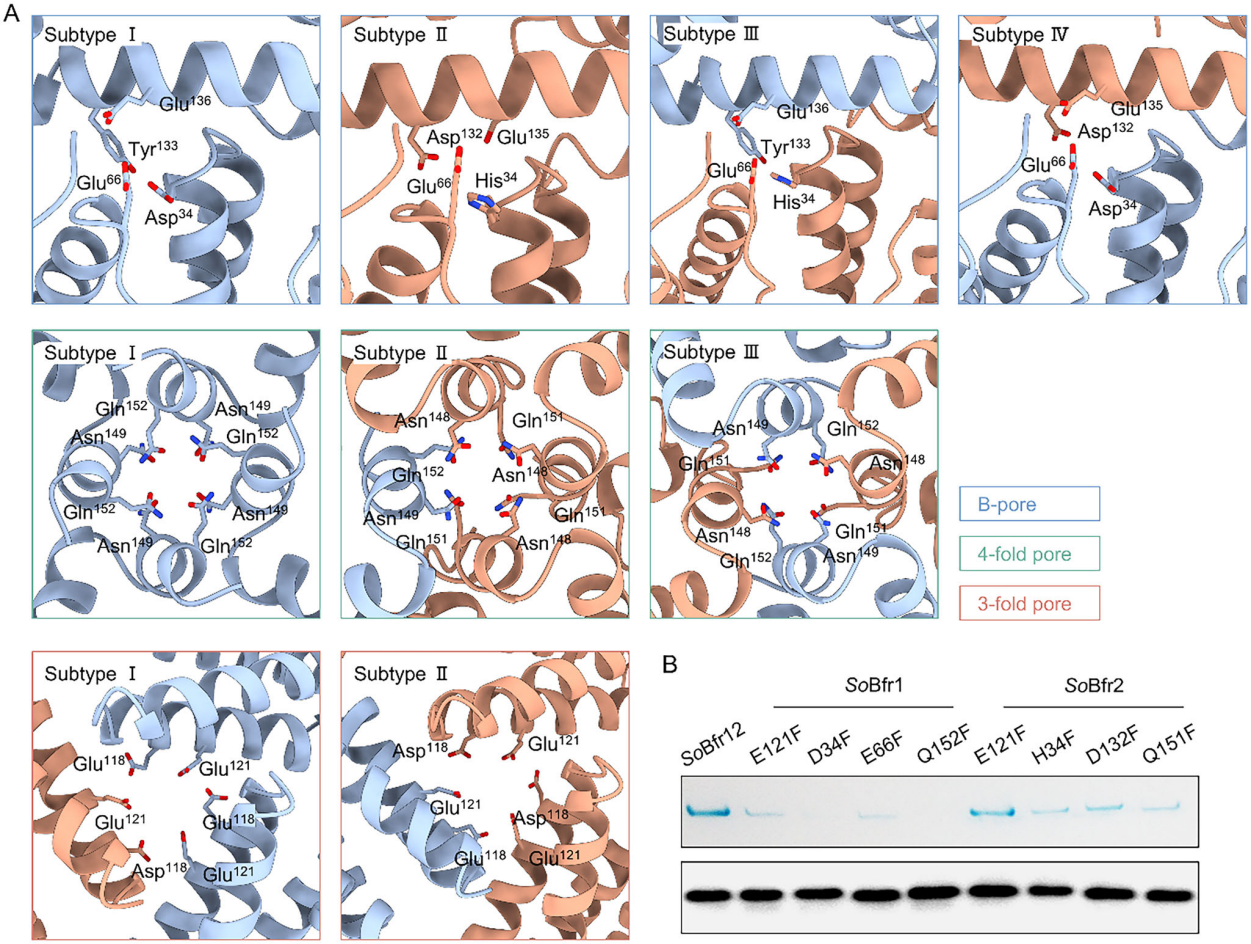
Close‐up views of the 4‐fold, 3‐fold, and B‐pores of *So*Bfr12 nanocages and their effect on iron storage. A) The Close‐up view of the 9 subtypes B‐pores, 4‐fold pores, and 3‐fold pores, composed of *So*Bfr1 (steel blue) and *So*Bfr2 (salmon) two subunits in different quantitative ratios. Negatively charged/hydrophilic residues near these pores are marked. Four subtypes of B‐pores are in the blue box, three subtypes of 4‐fold pores are in the green box, and two subtypes of 3‐fold pores are in the orange box. B) Iron storage ability of *So*Bfr12^WT^ and its variants. The top and bottom panels are Native‐PAGE iron staining and SDS‐PAGE Western blot results of *So*Bfr12^WT^ and its variants, respectively. *So*Bfr1 was tagged with a Strep‐II tag for Western blot. The cells used here were prepared in the same way as described in Figure 1D. The iron staining experiment was performed in three independent biological replicates for each condition (Figure S10, Supporting Information).

Like homo‐Bfrs,^[^ ^18^, ^19^, ^38^ ^]^ *So*Bfr12 comprises 38 channels through which ions traverse, including 24 B‐pores, six 4‐fold pores, and eight 3‐fold pores (**Figure** **3**). However, because all these pores are formed by two different homodimers in various combinations, the pores of the same type are not necessarily the same, resulting in 9 distinct subtypes: 4, 3, and 2 subtypes for B‐, 4‐fold, and 3‐fold pores, respectively. Notably, the numbers of pores in these subtypes vary substantially, from 1 for subtype I of 3‐fold pores to 8 for both subtypes III and IV of B‐pores (Figure 3). The B pores are characterized by one dimer vertically attaching to a subunit of another dimer with the negatively charged residues, including Asp^34^/His,^34^ Glu,^66^ Glu^135/136^ and Asp^132^ at the interfaces, and their subtypes have ratios of *So*Bfr1 to *So*Bfr2 in 3:0, 0:3, 1:2 and 2:1 (**Figure** **4**A). Remarkably, the interior surfaces of B‐pores, especially subtype I (3:0), are rich in negatively charged residues, and this feature likely facilitates the uptake of positively charged Fe^2+^ ions (Figure 4A; Figure S5 and Table S2, Supporting Information). Each 4‐fold pore is formed by the C‐terminal short helices of four subunits with the conserved residues of Asn^148^ and Gln^151^ from *So*Bfr1 or Asn^149^ and Gln^152^ from *So*Bfr2 protruding toward the pore cavity (Figures 3 and 4A; Table S2, Supporting Information). Three subtypes of the 4‐fold pores are defined by ratios of *So*Bfr1 to *So*Bfr2 in 4:0, 1:3, and 1:1 (Figure 3). Analysis of the surface electrostatic potential and hydrophobicity revealed that the subtype I 4‐fold pore formed by *So*Bfr1 only has a highly negative and hydrophilic pathway between the FC to the pore, along which Fe^2+^ ions move (Figures S5 and S6A,B, Supporting Information). In each subtype, a Na^+^ ion may coordinate with the conserved residues, likely due to the presence of sodium in the buffers and supported by previous studies on related Bfrs (PDB ID: 6P8K, 6P8L) (Figure S7A, Supporting Information). The 3‐fold pores, comprising two subtypes with ratios of *So*Bfr1 to *So*Bfr2 in 2:1 and 1:2, are formed by the helix‐loop‐helix (α3‐L4‐α4) regions of three subunits, with six conserved residues of Glu^118^ and Glu^121^ from *So*Bfr1 or Asn^118^ and Glu^121^ from *So*Bfr2 surrounding the pore cavity (Figures 3 and 4A; Table S2, Supporting Information). The distances from the central points of B‐, 4‐fold, and 3‐fold pores to the FC of *So*Bfr1 are 16.9 Å, 31 Å, and 23.3 Å, respectively (Figure S8, Supporting Information). Conceivably, compared to homo‐Bfrs, hetero‐Bfrs likely exhibit variations in preference and efficacy when Fe^2+^ ions or other ions enter and exit the nanocage through them. Based on these pores, the *C*2 symmetry of *So*Bfr12 is defined by the two‐fold axis connecting one subtype III 4‐fold pore and one subtype I 4‐fold pore (Figure 2B), which passes through the core.

The pronounced impacts were observed from (D/H)34F, E66F, D132F, and Q(151/152)F, residues in proximity to the B‐pores and 4‐fold pores, suggesting that the 4‐fold and B‐pores are more heavily involved in Fe^2+^ entry as well as iron oxidation and mineralization. More importantly, by pair‐wise comparison, reduction in iron levels caused by the same mutations (or at equivalent residues) in *So*Bfr1 was more drastic than in *So*Bfr2, indicating that *So*Bfr1 plays a substantially larger role in Fe^2+^ entry, iron oxidation, and mineralization than *So*Bfr2 (Figure 4B). This difference is significant because the numbers of *So*Bfr1 in the subtypes of 4‐fold pores and B‐pores vary (Figure 3), suggesting that the contributions of different subtypes of these pores to iron incorporation likely differ.

### 2.4 Functional Differentiation of *So*Bfr1 and *So*Bfr2

**Figure 5:**
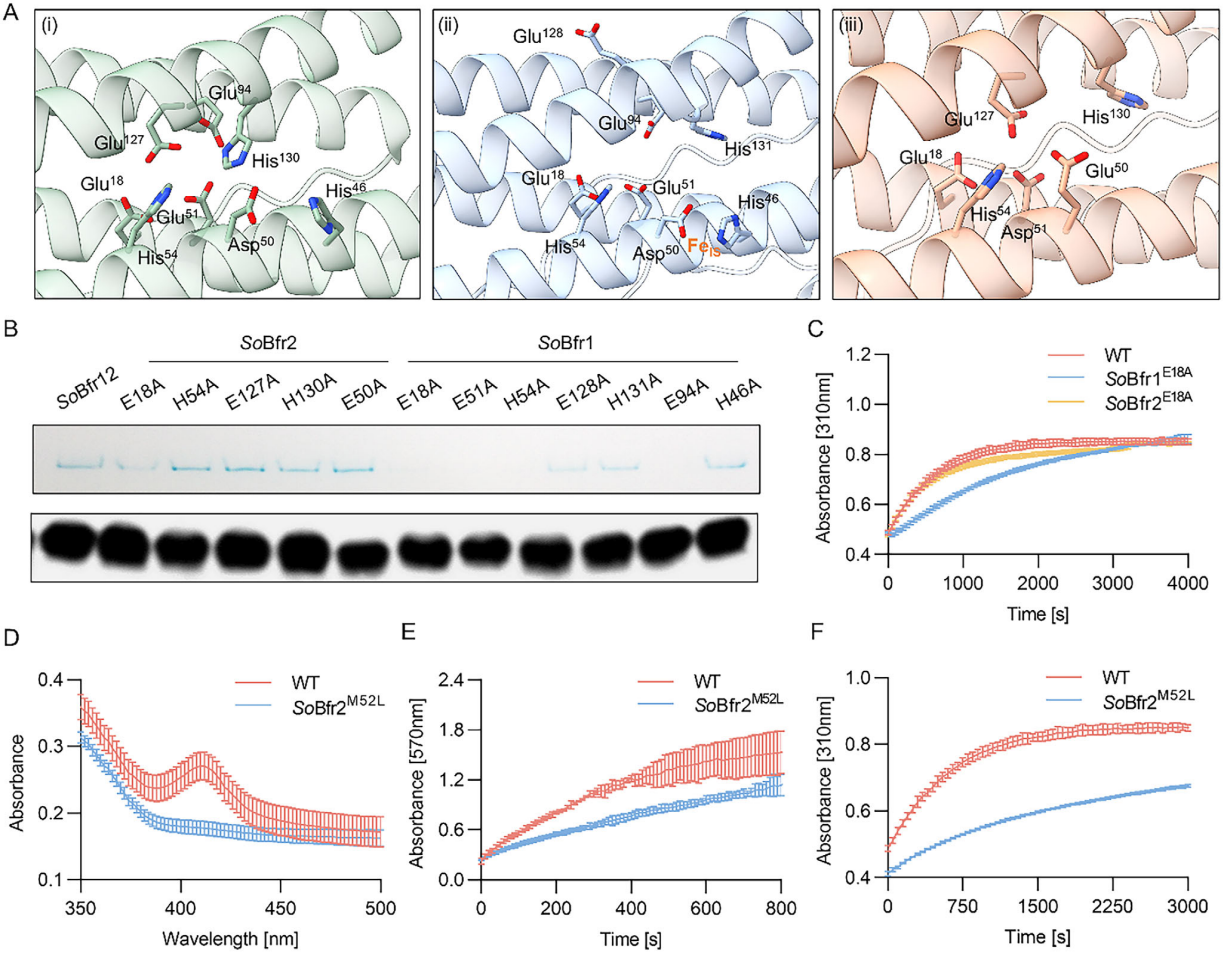
Characterizations and roles of the FCs and heme in *So*Bfr12. A) Close‐up views of the FCs in *So*Bfr12 and homo‐Bfr. From left to right, the FC of *Pa*Bfr (i), *So*Bfr1 (ii), and *So*Bfr2 (iii). Each homodimer possesses an FC embedded in the four‐helix bundle. The locations of the supposed iron ions (Fe_1_ and Fe_2_) in the FCs are shown as orange spheres, coordinated by Glu (Asp) and His residues. B) iron storage ability of *So*Bfr12^WT^ and its variants. The top panel shows the iron staining of *So*Bfr12^WT^ and its variants by Native‐PAGE. The bottom panel displays the expression of *So*Bfr12^WT^ and its variants by SDS‐PAGE Western blot. *So*Bfr1 was tagged with a Strep‐II tag for Western blot analysis. Complementation was carried out with a vector containing an IPTG‐inducible promoter Ptac. The results shown were obtained using 0.5 mmol IPTG. C) Iron oxidation progress curves of *So*Bfr12^WT^ and its variants (*So*Bfr1 E18A and *So*Bfr2 E18A), where the oxidation of Fe^2+^ to Fe^3+^ was monitored by an increase in absorbance at 310 nm. D) Absorption spectra of *So*Bfr12^WT^ and its variant *So*Bfr12^M52L^. The absorption peak ≈400 nm is attributed to the heme group. E) Comparison of the release of mineralized iron from *So*Bfr12^WT^ and its variant *So*Bfr12^M52L^, where the released iron was measured as Fe^2+^‐ferrozine complex at 570 nm. F) Iron oxidation curve of *So*Bfr12^WT^ and its variant *So*Bfr12^M52L^, where the oxidation of Fe^2+^ to Fe^3+^ was monitored by an increase in the absorbance at 310 nm. All data in (C–F) are mean ± SD (*n*  =  3 independent experiments). The iron staining experiment was performed in three independent biological replicates for each condition (Figure S10, Supporting Information).

In the FCs of *P. aeruginosa* homo‐Bfr (*Pa*BfrB, PDB ID: 4TOH), two iron atoms (Fe_1_ and Fe_2_) are coordinated by residues Glu,^18^ Glu,^51^ Glu^94^, Glu^127^, His,^54^ and His^130^, and the interior iron binding site (Asp^50^ and His^46^) is located at a distance from the FC (**Figure** **5**A).^[^ ^3^ ^]^ Not surprisingly, although heme‐free *So*Bfr1 and heme‐binding *So*Bfr2/*Pa*BfrB are highly similar in structure, their FCs differ from each other significantly (Figure 5A). Additionally, we also observed some differences in the FCs of *So*Bfr2 and *Pa*BfrB (Figure 5A). In the FCs of *So*Bfr12, two iron atoms (Fe_1_ and Fe_2_) are supposed to be coordinated by conserved residues Glu,^18^ Glu,^51^ Glu^94^, and His^54^ of *So*Bfr1 and by Glu,^18^ Asp,^51^ Glu^127^, and His^54^ of *So*Bfr2 (Figure 5A). Evidently, Glu^128^ of *So*Bfr1 is toward the exterior of the FC, whereas its counterparts in *So*Bfr2 and *Pa*BfrB adopt an opposite orientation (Figure 5A). In the case of His^131^, another residue stabilizing the two iron ions in the FCs of *Pa*BfrB,^[^ ^3^ ^]^ its counterparts swing away from the iron‐binding pocket in both *So*Bfr1 and *So*Bfr2. This difference can be readily explained by the fact that the residue takes the “gate closed” conformation in iron‐soaked *Pa*BfrB, whereas it is in the “gate open” conformation because non‐iron‐soaked *So*Bfr12 was used for Cryo‐EM in our study.^[^ ^3^ ^]^ Intriguingly, the interior iron‐binding site residues (Asp^50^ and His^46^) of *So*Bfr1 but not of *So*Bfr2 exhibit conformation identical to that of *Pa*BfrB (Figure 5A). More surprisingly, *So*Bfr2 employs Val and Lys in place of Glu and His at residues 94 and 46, respectively (Figure 1B), which are also conserved in *Pa*BfrB.^[^ ^27^, ^29^ ^]^ Given that Glu^94^ plays a critical role in stabilizing two iron atoms, its substitution implies that the FCs of *So*Bfr2 may largely lose the ability to oxidize iron, and therefore *So*Bfr1 is primarily responsible for iron oxidation. Particularly, His,^46^ His^131^ and Glu^128^ of *So*Bfr1 adopt opposite orientation in *So*Bfr1 compared to their counterparts in *So*Bfr2 and *Pa*BfrB, suggesting that these residues may not be important for iron storage in *So*Bfr12.

Analysis of the cryo‐EM map revealed two apparent low‐occupancy peaks at the FC of *So*Bfr1, and a single low peak adjacent to Glu^18^ in *So*Bfr2. These observations suggest that *So*Bfr1 may play a critical role in the ferroxidase activity of the *So*Bfr12 nanocage (Figure S7B,C, Supporting Information). In order to prove this, site‐directed mutagenesis was first applied to assess the impacts of most of the conserved residues at the FCs of *So*Bfr1 and *So*Bfr2 on iron incorporation capability. When expressed at similar levels, the resulting variants under test exhibited varying influence on the iron content of *So*Bfr12 (Figure 5B; Figures S9 and S10A, Supporting Information). Consistent with the observation presented in Figure 4B, *So*Bfr1 is substantially more sensitive than *So*Bfr2 to the mutations at the same/equivalent residues with respect to iron storage activity (Figure 5B; Figures S9 and S10A, Supporting Information), reinforcing that *So*Bfr1 dictates iron oxidation and mineralization. Apparently, only E18A out of representative variants appears to be critical for both homodimers, which is consistent with the results determined by inductively coupled plasma‐mass spectrometry (ICP‐MS) analysis (Figure S11A–C, Supporting Information). Consistently, the complex assembly of all mutants under examination appeared to be comparable to that of the WT (Figures S9 and S11B,C, Supporting Information). While all other *So*Bfr2 variants under test behave no observable difference compared to WT, their counterparts of *So*Bfr1, except for *So*Bfr1^H46A^ are clearly important for the activity (Figure 5B). Moreover, given that Glu^94^, which is missing in *So*Bfr2 but conserved in *Pa*BfrB, is essential for iron storage, we propose that this residue may significantly contribute to the difference in iron storage activity between *So*Bfr1 and *So*Bfr2. Furthermore, the mutation of His,^46^ His^131^ and Glu^128^ of *So*Bfr1 did not cause noticeable alterations in the iron staining bands (Figure 5B), confirming that they are not important for iron storage as suggested above.

The notion that *So*Bfr1 rather than *So*Bfr2 plays a dominant role in the ferroxidase activity of *So*Bfr12 was further supported by the kinetic analysis of iron oxidation and iron release. During iron oxidation, compared to *So*Bfr12^WT^, while *So*Bfr2^E18A^ showed a curve only marginally different, *So*Bfr1^E18A^ showed drastically slow kinetics, especially at the early stage (Figure 5C). To investigate the role of the heme molecules in *So*Bfr12, a heme‐free variant (*So*Bfr2^M52L^) was constructed by substituting the axial residue methionine with leucine, and the resulting *So*Bfr2^M52L^ became unable to bind heme as the characteristic peak of heme ≈410 nm was absent (Figure 5D). Compared to *So*Bfr12^WT^, *So*Bfr2^M52L^ displayed an obvious reduction in not only the releasing rates but also the oxidation rates, indicating that the heme molecule participates in both iron oxidation and reduction processes (Figure 5E,F).

### 2.5 Functional Comparison Between Homo‐ and Hetero‐Bfrs

**Figure 6:**
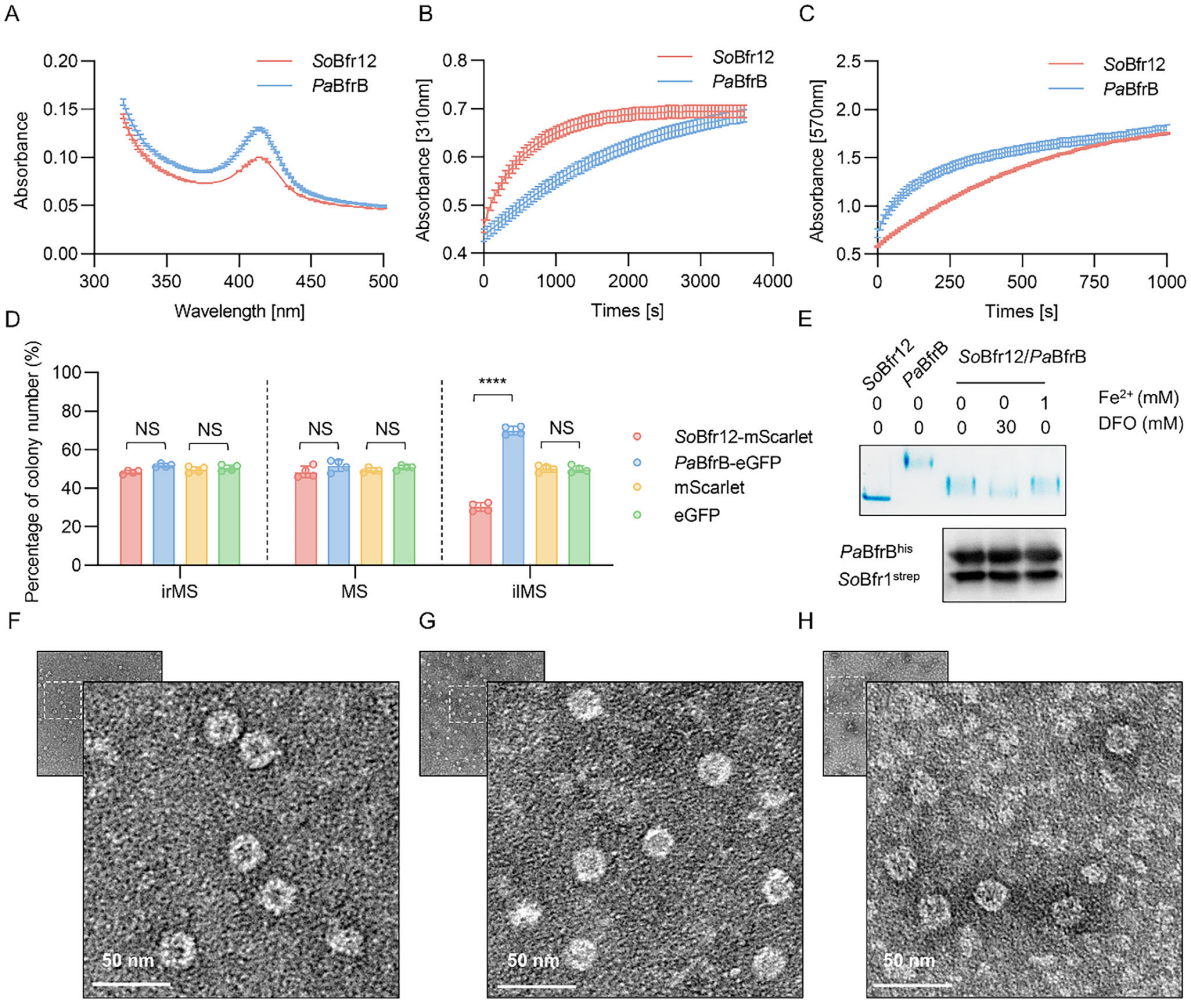
Biochemical characterization and growth competitiveness analysis of *So*Bfr12 and *Pa*BfrB. A) Absorption spectra of *So*Bfr12 and *Pa*BfrB. The absorption peak ≈400 nm is attributed to the heme group. B) Iron oxidation curve of *So*Bfr12 and *Pa*BfrB. The oxidation of Fe^2+^ to Fe^3+^ was monitored by an increase in absorbance at 310 nm. C) Comparison of the release of mineralized iron from *So*Bfr12 and homo‐Bfr. The released iron was measured as Fe^2+^‐ferrozine complex at 570 nm. D) Proportions of mScarlet‐ and eGFP‐labeled colonies under different iron conditions. irMS, iron‐rich defined medium MS (containing 1 mmol FeSO_4_); MS, normal medium MS; ilMS, iron‐limited defined medium MS (containing 30 mmol membrane‐impermeable iron‐chelating siderophore DFO). E) Iron loading ability and iron incorporating efficiency of *So*Bfr12/*Pa*BfrB under different iron conditions by Native PAGE iron staining (Top). Expression of co‐expressed *So*Bfr12 and *Pa*BfrB in Δ*bfr12* strains under different iron conditions by Western blot analysis (Bottom). F–H) Representative TEM images of *So*Bfr12, *Pa*BfrB, and *So*Bfr12/*Pa*BfrB, respectively. Data in (A–C) are mean ± SD (*n*  =  3 independent experiments), and in (D) are mean ± SD (*n*  =  4 independent experiments). The significant difference was evaluated by two‐tailed unpaired Student's *t*‐test, *, *p* < 0.05; **, *p* < 0.01; ***, *p* < 0.001; ****, *p* < 0.0001 in (D). The iron staining experiment was performed in three independent biological replicates for each condition (Figure S10A, Supporting Information).

To address whether hetero‐Bfr differs from homo‐Bfr in iron storage activity, we compared iron oxidation and release kinetics of *So*Bfr12 and *Pa*BfrB. Not surprisingly, *So*Bfr12 showed a heme content lower than *Pa*BfrB (**Figure** **6**A; Figure S12A,B, Supporting Information). In line with the finding that heme‐free *So*Bfr1 is more efficient in iron oxidation, kinetic analysis revealed that *So*Bfr12 exhibited a faster oxidation rate of Fe^2+^ than *Pa*BfrB (Figure 6B). In contrast, *Pa*BfrB appeared more effective in reducing Fe^3+^ (Figure 6C).

The discrepancy in iron oxidation and reduction ability between hetero‐Bfr and homo‐Bfr prompts us to examine whether this is of physiological significance. To this end, growth competition between Δ*bfr12* strains expressing *So*Bfr12 and *Pa*BfrB, along with a fluorescent protein eGFP or mScarlet for easy identification, was performed (Figure S13A, Supporting Information). The relative fitness of these two strains in iron‐rich (containing 1 mmol FeSO_4_), normal, and iron‐limited (containing 30 mmol membrane‐impermeable iron‐chelating siderophore DFO) defined medium MS (irMS, MS, and ilMS, respectively) was determined after approximately three generations. While both strains retained the initial ratio when grown in irMS and MS, Δ*bfr12*/p*PabfrB* cells overgrew Δ*bfr12*/p*Sobfr12* significantly in ilMS, making up 65% of the population (Figure 6D), suggesting that homo‐Bfr confers an advantage in fitness to cells under iron‐limited conditions.

We then attempted to address whether subunits of *So*Bfr12 and *Pa*BfrB can assemble functional nanocages in the cell. All three subunits, *So*Bfr1, *So*Bfr2, and *Pa*BfrB, were co‐expressed in the Δ*bfr12* strain (Figure 6E; Figures S10B and S13B,C, Supporting Information). Interestingly, in Native PAGE analysis, the purified *Pa*BfrB complexes migrated not only significantly slower than *So*Bfr12, but also in a smear band, suggesting that the PaBfrB nanocage formed a higher‐order oligomer (≈880 kDa) (Figure 6E; Figure S14, Supporting Information). From cells expressing *So*Bfr1, *So*Bfr2, and *Pa*BfrB together, we observed a similar smear band migrating faster than *Pa*BfrB but slower than *So*Bfr12. This band was similar in intensity in cells grown in irMS and MS but less intensive in cells grown in ilMS, in accord with the proposal that Bfrs release iron to maintain iron homeostasis when iron is scarce.^[^ ^19^, ^40^ ^]^ Transmission electron microscopy (TEM) revealed that the Bfr particles in these cells have a cage‐like morphology with a size similar to that of *So*Bfr12 (Figure 6F–H), supporting that they all comprise 24 subunits. Thus, we conclude that *Pa*BfrB subunits, presumably in homodimers, can be assembled into the nanocages together with *So*Bfr1 and *So*Bfr2.

### 2.6 Hetero‐Bfrs May Evolve Out Through Multiple Routes

**Figure 7:**
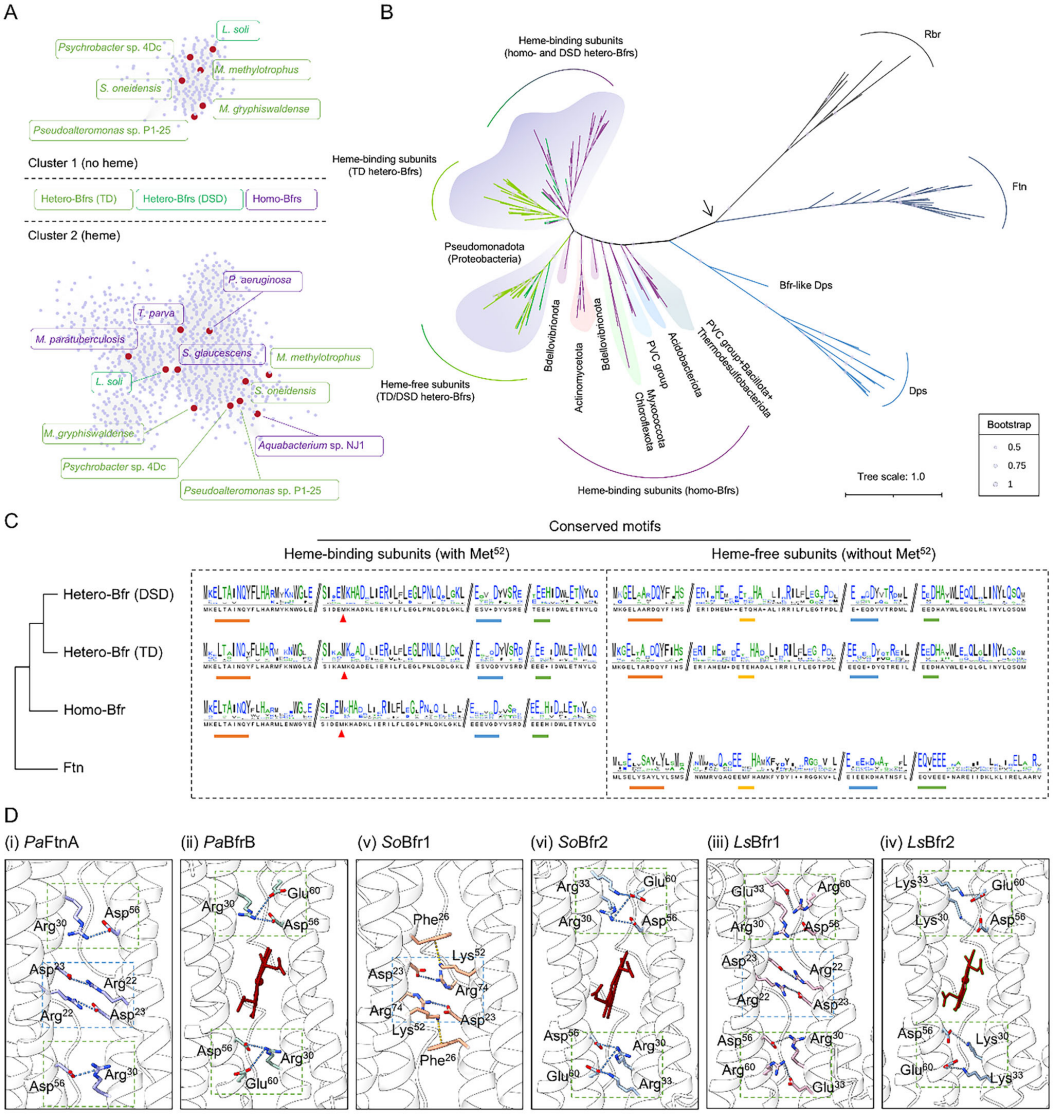
Phylogenetic analysis determines the emergence of Bfr subfamily members. A) SSN of Bfrs including homo‐Bfrs, hetero‐Bfrs (DSD), and hetero‐Bfrs (TD). Shown is the distribution of Bfrs with or without heme‐binding sites. The representative Bfrs from Figure 1 are marked as red dots. Bacteria containing homo‐Bfrs, hetero‐Bfrs (DSD), and hetero‐Bfrs (TD) are in purple, green, and light green boxes, respectively. B) Phylogenetic relationships of diversified subfamilies of Bfr, Dps, Ftn, and Rbr in bacteria. Circle sizes on branches indicate the support value calculated using a fast likelihood‐based method aLRT. The arrow points to the presumed base of the tree, which lies in proximity of the divergence between Rbr and Ftn clades. Each clade is coloured according to the protein composition: Rbr, grey; Ftn, cyan; Dps, blue; homo‐Bfr, purple; hetero‐Bfr (DSD), green; hetero‐Bfr (TD), light green. C) Conservation of the ferritin‐like domain of the diversified Bfr and Ftn subfamilies. The red arrow points to the Met^52^ residues that bind the heme molecules. The strip lines correspond to the four conserved ferritin‐like domains. D) Close‐up views of the interactions between two subunits from *Pa*FtnA (PDB ID: 3R2H), *Pa*BfrB (PDB ID: 4TOH), *So*Bfr1 (PDB ID: 9IIG), *So*Bfr2 (PDB ID: 9IIG), *Ls*Bfr1 and *Ls*Bfr2 (predicted by Alphafold3) dimers. The salt bridges between two charged residues are colored in light steel blue. The π‐cation interactions between aromatic rings and cationic side chains are colored in gold.

It is believed that the members of all three subfamilies within the ferritin family evolve out from a common ancestor.^[^ ^9^ ^]^ To quantifiably delineate Bfr subfamily members, we generated a sequence similarity network (SSN) using EFI toolset,^[^ ^41^, ^42^ ^]^ which is independent of phylogenetic evolutionary inference (Figure S15, Supporting Information). In this analysis, sequences with high identity (≥ 50%) collapsed into nodes, which were assembled into a large number of clusters. It is immediately evident that all members of Ftn, Bfr, and Dps subfamilies are sufficiently different from those of other subfamilies so that most, if not all, of the clusters comprise proteins from the same subfamily only (Figure S15, Supporting Information). This is also the case with the heme‐binding and heme‐free subunits (**Figure** **7**A). Bfr cluster 1 consists of heme‐binding subunits from both homo‐Bfrs and hetero‐Bfrs, whereas cluster 2 comprises heme‐free subunits, exemplified by the subunits from all representative Bfrs presented in Figure 1A (Figure 7A).

To elucidate the evolutionary relationships of the subfamilies, a maximum‐likelihood phylogenetic tree was constructed (Figure 7B). In this tree, three clades comprising Ftn, Dps, and Bfr subfamilies, along with a clade for ferritin‐like rubrerythrins (Rbr), are separated well from others, indicating that sequence conservation within each subfamily is sufficiently high to define a phylogenetic branch. Given that Rbr is considered to be more closely related to the common ancestor of the ferritin‐like superfamily,^[^ ^9^, ^17^ ^]^ the presumed base of the tree lies in the proximity of the divergence between Rbr and Ftn clades. According to this base, it is apparent that both Ftn and Dps proteins appeared earlier than Bfrs. It should be noted that a small number of proteins, called the Bfr‐like (because of its Bfr‐like intrasubunit FC) 12‐mer Dps proteins,^[^ ^9^ ^]^ are found in the Dps branch, suggesting that they have more likely evolved as canonical Dps. Along the evolutionary pathway in the tree, homo‐Bfr appeared before its hetero‐Bfr counterpart (Figure 7B), consistent with the idea that hetero‐Bfrs, at least some of them, evolve out from a homo‐Bfr through gene duplication. The homo‐Bfr‐only clade comprises multiple subclades based on heme‐binding Bfr subunits from diverse bacteria, suggesting that these proteins have a relatively low sequence conservation. This characteristic appears to be also true with the homo‐ and hetero‐Bfr mixing clade that is also based on heme‐binding subunits. In contrast, heme‐free subunits form a distinct clade (Figure 7B). Importantly, the branches of the heme‐binding and heme‐free subunits from TD‐type hetero‐Bfrs are closely related, supporting that they result from tandem duplication of a homo‐Bfr gene. However, the evolutionary linkages of the two subunits of DSD‐type hetero‐Bfrs seem uncertain, given that their corresponding branches are distantly separated from each other.

To explore other possible sources for subunits of DSD‐type hetero‐Bfrs, a comparative analysis of the Bfr and Ftn conserved sequences was conducted, focusing on four signature motifs, E‐X_6_‐Y (in helix α1), ExxH (in helix α2), E‐X_6_‐L/I (in helix α3) and QxxE/ExxH (in helix α4).^[^ ^9^ ^]^ An apparent feature of all heme‐binding subunits is the loss of the ExxH (in helix α2) motif, a result of adaptation to heme binding (Figure 7C). In terms of the other three motifs, the heme‐binding subunits from both types of hetero‐Bfrs are similar to those from homo‐Bfrs. The four conserved motifs were also observed in heme‐free subunits of hetero‐Bfrs, and importantly, they are highly similar to those within Ftns (Figure 7C).

Furthermore, a structural comparison between the interacting surfaces of Ftn (*Pa*FtnA, PDB ID: 3R2H) and Bfrs (*Pa*BfrB, *So*Bfr1 and *So*Bfr2) dimers revealed that in the *Pa*FtnA dimer, the interaction between the two subunits is mediated by four salt bridges, involving two Arg^30^‐Asp^56^ pairs and two Arg^22^‐Asp^23^ pairs (Figure 7D). In the heme‐free *So*Bfr1 dimer, only two salt bridges are formed between Arg^74^‐Asp^23^ pairs, resembling those between Arg^22^‐Asp^23^ pairs within *Pa*FtnA, and additional bonding force is provided by π‐cation interactions between two Phe^26^‐Lys^52^ pairs (Figure 7D). In the case of the heme‐binding Bfr dimers (from *Pa*BfrB and *So*Bfr2), the formation of salt bridges is substantially influenced by heme. The *Pa*BfrB dimer has four salt bridges formed between Arg^30^‐Glu^60^ pairs as well as Arg^30^‐Asp^56^ pairs, whereas the heme‐binding *So*Bfr2 dimer utilizes six salt bridges, between Arg^30^‐Glu^60^ pairs, Arg^33^‐Glu^60^ pairs, and Arg^33^‐Asp^56^ (Figure 7D). In addition, two subunits of DSD‐type hetero‐Bfr from *L. soli*, *Ls*Bfr1 and *Ls*Bfr2, were structurally predicted by AlphaFold3 and subjected to the same analyses. With respect to the interactions between two subunits of both homodimers, while heme‐binding *Ls*Bfr2 resembles *Pa*BfrB and *So*Bfr2, heme‐free *Ls*Bfr1 is highly similar to *Pa*FtnA but substantially different from *So*Bfr1. Combining similarities observed in the conserved motifs (Figure 7C), these results suggest a possibility that the heme‐free subunit of DSD‐type hetero‐Bfrs may evolve from Ftn directly.

## 3 Discussion

Metal ions, iron in particular, serve as crucial cofactors for a range of enzymatic reactions and are indispensable for the growth and diverse metabolic processes of microorganisms.^[^ ^43^, ^44^ ^]^ Iron‐storing Bfrs constitute a major subfamily of the Ftn family and play a major role in maintaining intracellular iron concentrations within a relatively stable range in a large portion of bacteria. Bfr nanocages can be found in homo‐ or hetero‐multimeric form, assembled from 24 identical subunits or from two distinct subunits, 12 each.^[^ ^3^ ^]^ While the structures and mechanisms of homo‐Bfrs are well understood,^[^ ^19^, ^23^, ^26^, ^45^ ^]^ hetero‐Bfrs remain underexplored. In this study, we aim to elucidate the structural, functional, physiological, and evolutionary characteristics of the TD‐type hetero‐*So*Bfr12, which consists of two Bfr subunits in equal proportions. All data from the cryo‐EM structure determination and biochemical and physiological analyses converge to support that hetero‐Bfrs are fundamentally different from homo‐Bfrs in structural details, including highly diverse pores, functionally differentiated FCs, and heme‐binding sites, which amount to substructures specialized for oxidation, mineralization, and reduction of iron ions. In addition, evolutionary analyses suggest that TD‐type hetero‐Bfrs likely arose from the duplication of a homo‐Bfr, while DSD‐type ones may have originated from either a Ftn or a homo‐Bfr through gene duplication.

A single‐particle cryo‐EM approach was used to determine the 2.6 Å‐resolution structure of *So*Bfr12 (PDB ID: 9IIG). Although this hetero‐Bfr exhibits highly conserved nanocage architecture as its homo counterpart, the heterogeneity of the subunits introduces substantial differences in the characteristics of these two types of nanocages. First, instead of the *O* symmetric subunit arrangement in homo‐Bfrs,^[^ ^18^, ^19^, ^38^ ^]^ hetero‐Bfrs adopt a *C*2 symmetry. One of the most important consequences caused by this symmetry is the changes in configurations of pores through which various ions pass. In hetero‐Bfrs, B‐pores, 4‐fold pores, and 3‐fold pores can be further divided into four, three, and two different subtypes, respectively, in contrast to homo‐Bfrs, in which the pores of the same type could not be differentiated from one another.^[^ ^18^, ^19^, ^38^ ^]^ Conceivably, the ions, especially Fe^2+^, may prefer certain pores as entry channels to others. In addition, the FCs of the hetero‐Bfrs and homo‐Bfrs differ significantly in terms of conformation and amino acid residues critical to iron oxidation. Compared to a heme‐binding subunit of both homo‐Bfrs and hetero‐Bfrs, residue Glu^128^ in the FC of the heme‐free subunit takes an opposite conformation, suggesting that the contribution of this residue to stabilize two irons is not likely to be significant.^[^ ^3^ ^]^ Moreover, we observed that His^131^ of both subunits of hetero‐Bfr, another residue involved in stabilizing irons, takes the “open gate” conformation, presumably because non‐iron‐soaked proteins were used in this study. Furthermore, a couple of residues, Glu^94^ and His,^46^ that contribute to iron oxidation are absent in the FC of the heme‐binding subunit, implying that the heme‐free subunit plays a predominant role in iron oxidation. As the heme‐free subunit is unable to carry out iron reduction, it is clear that the two subunits of hetero‐Bfrs have evolved to be specialized for iron storing and releasing. In addition, interaction forces, such as the salt bridges, within both heme‐binding and heme‐free dimers of hetero‐Bfrs are substantially altered compared to typical homo‐Bfrs and Ftns (Figure 7D), suggesting that evolution has shaped these proteins at multiple levels.

**Figure 8:**
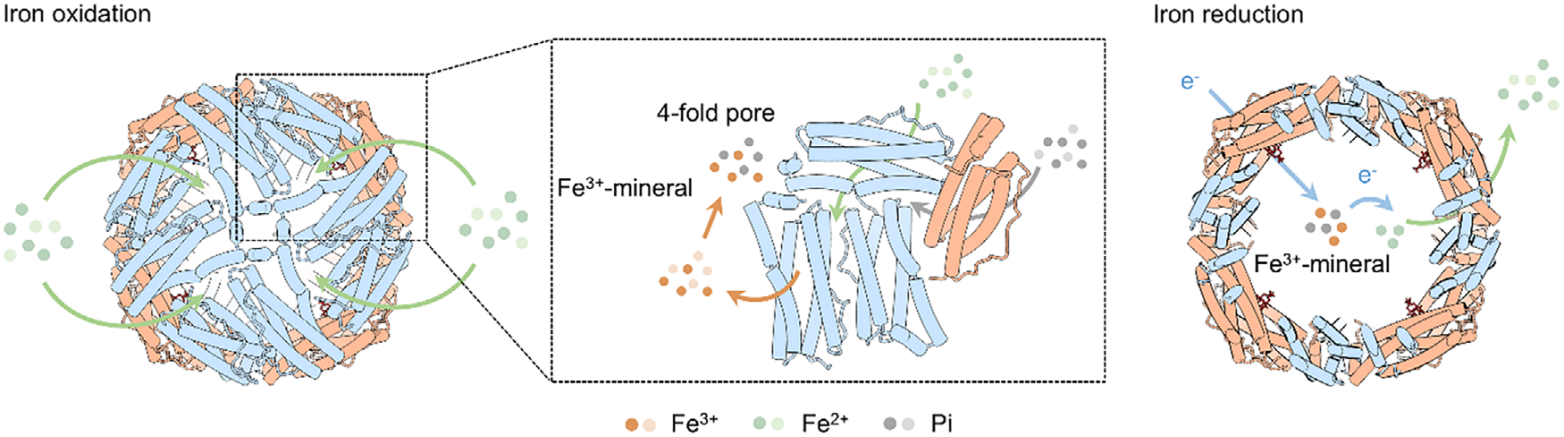
The proposed iron movement model within hetero‐Bfrs. In this model, the Fe^2+^ ions enter the spherical shell through the B‐pore composed of *So*Bfr1 subunits and are transported to the ferroxidase center of *So*Bfr1 to be further oxidized to Fe^3+^ ions. Subsequently, Fe^3+^ ions are directed to the 4‐fold pore for the initial biomineralization and form the iron‐phosphate mineral core. Heme groups from *So*Bfr2 dimers accept electrons from Bfd and transport them into the nanocage cavity along with the reduction of Fe^3+^ to Fe^2+^.

These characteristics of hetero‐Bfrs are supported by mutational analyses of key residues critical for the activity of pores and FCs. We showed that Fe^2^⁺ has a strong preference for B‐pores, especially subtype I ones. In contrast, the mutation (E121F) of the residue near the three‐fold pore exhibited the least effect on the changes in iron storage, implicating that this pore is probably associated with the entry of ions other than Fe^2+^. Ferroxidase activity of the heme‐binding dimer is significantly reduced compared to its heme‐free counterpart. Based on these data, we propose a model for iron entry, oxidation, and reduction in hetero‐Bfrs (**Figure** **8**). The initial entry of Fe^2+^ into the interior of the nanocage is primarily facilitated by subtype I B‐pores and then reaches the FCs for oxidation. Following oxidation, the Fe^3+^ moves from FC to the proximity of 4‐fold pores, particularly subtype I, through an acidic residue‐rich channel where mineralization begins.^[^ ^26^ ^]^ When needed, the heme group in the heme‐binding dimer is able to transfer electrons to Fe^3+^‐mineral, reducing and releasing iron. During the processes of iron storing and releasing, the heme‐free subunit dictates iron oxidation, while the heme‐binding subunit is responsible for iron reduction. In the context of this feature, hetero‐Bfr resembles mammal Ftn, which contains two chains evolved in a functionally divergent manner: the heavy (H)‐chain provides the FC, and the light (L)‐chain stabilizes the oligomerization and facilitates the iron influx into the nanocage.^[^ ^17^, ^46^, ^47^, ^48^, ^49^ ^]^

Given that the heme‐free subunit is rather specialized for iron oxidation, it is not surprising that hetero‐Bfr exhibits a greater iron oxidization ability than homo‐Bfr, which gains support from kinetic analyses in this study. On the other hand, hetero‐Bfr has a relatively weak iron reduction ability as it depends on heme. Interestingly, the assessment of these two complexes on overall fitness with growth competition revealed that homo‐Bfr displays an advantage over hetero‐Bfr in supporting growth, particularly under iron‐deficient conditions. We speculate that the difference may mainly result from the slow iron release mediated by heme‐binding subunits. *Shewanella* thrives in redox‐stratified environments, where iron, especially soluble ferrous ion, is relatively abundant, and are substantially more sensitive to ROS than model bacteria, such as *E. coli*.^[^ ^17^, ^50^, ^51^ ^]^ Over the course of evolution, iron oxidation capacity is enhanced in these bacteria such that they can rapidly reduce intracellular iron concentrations to survive through oxidative stress caused by excess iron. Apparently, this adaptation is at the cost of compromised iron release. Despite these differences, the heme‐free subunit of hetero‐Bfr remains highly similar to its heme‐binding subunits, either from hetero‐Bfr or homo‐Bfr. Additional expression of the heme‐binding subunit of homo‐Bfr in cells having hetero‐Bfr results in the formation of hetero‐Bfr nanocages assembled from all three subunits. The subunit composition of this complex seemingly depends on their expression levels. Similar scenarios have been reported in *P. aeruginosa* and mammals. While mammal Ftn contains heavy (H) and light (L) chains that are assembled in various proportions naturally, hetero‐oligomeric 24‐mer Bfrs varying in the subunit composition can be assembled from forcibly expressed *P. aeruginosa* FtnA and BfrB subunits.^[^ ^47^, ^48^, ^52^ ^]^ All of these data conclude that the members of the Bfr subfamily, and even of the Ftn subfamily, not only are closely related in phylogeny but also highly conserved in function and structure. We also made attempts to answer how hetero‐Bfrs evolve from the common ancestor of the ferritin family. In the phylogenetic tree of the ferritin family, the Bfr subfamily clades are expectedly away from the presumed base of the tree, which is in proximity to the divergent point of the Rbr family and the Ftn subfamily (Figure 7B). It is also clear that homo‐Bfrs appeared earlier and are more widely distributed than hetero‐Bfrs, indicating that this type of Bfr is rather stable, probably withstanding greater selective pressure and better adapt to the changing environment. In line with the finding that TD‐derived genes are more stable than DSD‐derived genes,^[^ ^53^ ^]^ TD‐type hetero‐Bfrs are found to be restricted to a relatively small group of bacteria, mostly Proteobacteria, and some archaea, whereas DSD‐type hetero‐Bfrs have a broader distribution. After tandem duplication of a homo‐Bfr gene, the two descendants appear to evolve independently of each other and either can be turned into a heme‐free subunit, leading to comparable distribution of the opposite orders of the two *bfr* genes for TD‐type hetero‐Bfrs. Clearly, all of these data converge on the conclusion that TD‐type hetero‐Bfrs evolve from homo‐Bfrs. In the case of DSD‐type hetero‐Bfrs, however, the evolution relationships appear complex. Although DSD is a major type of gene duplication from prokaryotes to eukaryotes, the underpinning mechanisms remain unclear.^[^ ^53^, ^54^ ^]^ The high similarity of the conserved ferritin‐like motifs between Ftns and the subunits of DSD‐type hetero‐Bfrs suggests a possibility that both subunits may be a result of dispersed duplication of a *ftn* gene. The similar interaction patterns observed in Ftn and the dimers of the heme‐free subunit of DSD‐type hetero‐Bfr (Figure 7D) strongly support the idea that this subunit comes directly from Ftn. One may envision that *ftn* duplication occurred early in a DSD‐type manner, even possibly before the advent of homo‐Bfr and multiple times. Among the resulting homologous proteins, one eventually acquired heme and became homo‐Bfr in some prokaryotes, and in some others, in addition to one that was converted to homo‐Bfr, another homolog remains heme‐free but develops sufficiently competitive ability to complex with the heme‐binding subunits of homo‐Bfr, resulting in hetero‐Bfr. Because the DSD‐type hetero‐Bfrs showed up much later than homo‐Bfrs we speculate that a long time is required for the heme‐free homolog to evolve out this complexation capability. This notion is clearly in line with recent findings that Bfr and Ftn form hetero‐oligomers rather than coexisting as homo‐oligomers in bacteria.^[^ ^52^, ^55^ ^]^ The crystal structure of the heterooligomeric *Acinetobacter baumannii* Bfr (*Ab*Bfr), composed of ≈39% *Ab*Ftn and 61% *Ab*Bfr subunits, reveals that Ftn homodimers harbor FC, while Bfr homodimers lack FC but are capable of binding heme.

## 4 Experimental Section

### Protein Expression and Purification

Bacterial strains, plasmids, and the sequences of the primers used in this study can be found in Tables S4 and S5 (Supporting Information). *E. coli* and *S. oneidensis* were grown in Lennox broth (LB, Difco, Detroit, MI, USA) medium under aerobic conditions at 37 and 30 °C, supplemented with 50 µg mL^−1^ kanamycin, 15 µg mL^−1^ gentamicin, or 0.3 mmol 2,6‐daminopimelic acid when necessary. Recombinant *So*Bfr12 was overexpressed in *E. coli* [BL21(λDE3)] that was transformed with pET28a(+) plasmid. The region coding for *So*Bfr12^WT^ was amplified from *S. oneidensis* genomic DNA using primers pET‐28a(+)‐Bfr12^strep^‐F and pET‐28a(+)‐Bfr12^strep^‐R, and cloned into pET‐28a(+) plasmid (Supplementary Table S4). *So*Bfr12^WT^, *Pa*BfrB, and all *So*Bfr12^WT^ variants were produced in *E. coli* [BL21(λDE3)] as C‐terminal fusions to a Strep‐II tag and purified by strep‐affinity chromatography. The expression of the gene of interest was induced by IPTG. In summary, the overnight cultured cells were used to inoculate an LB medium (2 L) containing kanamycin and grown at 37 °C. After OD_600_ reached 0.6–0.8, the cultures were added with 0.5 mmol IPTG followed by incubation for 4–5 h. Cells were harvested by centrifugation at 5500 rpm, 4 °C, 20 min. The harvested cells were resuspended in 50 mL lysis buffer (20 mmol Tris‐HCl pH 8.0, 150 mmol NaCl, 5% glycerol supplemented with DNase I, and 1 mmol PMSF). Cells were disrupted with a cell disruptor equipped with a cooling system. Cell debris was removed by centrifugation at 15 000 rpm for 30 min. The supernatant was first purified by Strep‐tag affinity chromatography on a 5 mL PreCap STarm Streptactin (Changzhou Smart‐Lifesciences Biotechnology Co., Ltd). The elution fractions were concentrated and further purified by size‐exclusion chromatography on a Superdex 200 Increase 10/30 column (GE Healthcare Life Sciences). Fractions containing *So*Bfr12 were pooled for Cryo‐EM experiment, and the protein purity was evaluated by SDS‐PAGE and Native‐PAGE. Samples for data collection were further checked using TEM at 120 kV. The purification processes for *Pa*BfrB, *So*Bfr12/*Pa*BfrB, and *So*Bfr12 mutants followed identical protocols.

### Transmission Electron Microscopy

A volume of 3 µL protein solution was applied to a 300‐mesh carbon‐coated copper grid. After incubating for 30 s, the excess solution was soaked away using filter paper. The samples were subsequently stained three times with 2% (w v^−1^) uranyl acetate solution. Finally, the dried grids were observed with a Thermo Scientific Talos L120C transmission electron microscopy. For cryo‐EM sample preparation, a volume of a 3 µl purified *So*Bfr12 sample (1.7 mg mL^−1^) was applied to a holey carbon grid (Quantifoil R1.2/1.3, Au, 300 mesh). After 30 s, the grids were blotted for 6 s (blot force 5) at a humidity of 100% and 10 °C and plunge‐frozen in liquid ethane using a Vitrobot (FEI). Cryo‐EM images of *So*Bfr12 were acquired with an FEI 300 kV Titan Krios electron microscope equipped with a FEI Falcon4 direct electron detector + energy filter (FEI Selectris) at a nominal magnification of 130 000×. The defocus range was set between −1 and −1.5 µm, and the dataset was recorded with a total exposure dose of 50 e A^−2^. During the data processing, the EER upsampling factor was set to the default value of 2 during the import of the movie frames, which resulted in 8000 × 8000 images with an effective super‐resolution pixel size of 0.465 Å pixel^−1^. All images were processed with CryoSPARC 4.4.^[^ ^56^, ^57^ ^]^ A total of 6940 movies were motion‐corrected using Patch motion correction to generate average dose‐weighted micrographs. CTF estimation was conducted using Patch CTF. After this, the resulting data (with a pixel size of 0.465 Å pixel^−1^) were used for particle extraction and 2D/3D classification. In brief, particles were blob‐picked, yielding 1654539 particles, which were subjected to 2D classification to generate templates for template‐based picking. Then, 4931086 particles were template‐picked. 2D and 3D classifications were carried out to produce 736 353 particles for further 3D refinement. Nonuniform refinements were accomplished with C1 symmetry, resulting in a density map with a resolution of 2.62 Å.

### Model Building and Refinement

The AlphaFold3‐predicted structure was used as a reference model to fit into the corresponding density map using UCSF ChimeraX.^[^ ^58^ ^]^ The initial structure was manually modified in Coot.^[^ ^59^ ^]^ Through several iterative operations of manual adjustment in Coot and real‐space refinement in Phenix,^[^ ^60^ ^]^ the final model was obtained and deposited in the Protein Data Bank as entry 9IIG. All structural visualizations were generated using UCSF ChimeraX.^[^ ^58^ ^]^

### Site‐Directed Mutagenesis

Site‐directed mutagenesis was employed to generate *So*Bfr12 proteins carrying point mutations. The *bfr12* gene within the vectors used for expression and purification was subjected to modification by using a QuikChange II XL site‐directed mutagenesis kit (Stratagene) as described previously.^[^ ^61^ ^]^ After verification by sequencing, the resultant vectors were transferred into the relevant strains via conjugation.

### SDS‐PAGE, Native PAGE, and Western blot analysis

Conventional SDS‐PAGE was performed using slab gels consisting of a 15% acrylamide separating gel and a 5% stacking gel. For native PAGE, protein samples were separated using slab gels consisting of a 6% acrylamide gel with Tris‐Glycine electrophoresis buffer (25 mmol Tris, 192 mmol glycine, pH 8.3). Protein samples were mixed with native sample buffer, loaded into the gel, and electrophoresis was performed at 100 V for 1 h (at 4 °C). Proteins on the SDS‐PAGE and Native PAGE gels were then electrophoretically transferred to PVDF membrane (Millipore, Bedford, MA) according to the manufacturer's instructions (Bio‐Rad, Hercules, CA, USA). 5% BSA was used to block the membrane. The membrane was probed with a 1:10 000 dilution of a mouse monoclonal his‐tag or strep‐tag II antibody (Abbkine, Shanghai, China), followed by a 1:10 000 dilution of Goat anti‐mouse IgG‐HRP (horseradish peroxidase) (Beyotime, Beijing, China) and the signal was detected using a chemiluminescence Western blotting kit (Roche, Basel, Switzerland). Images were visualized with ChemiScope 6000 Imaging System (Clinx, Shanghai, China).

### Iron Mineralization and Iron Staining

Samples for assessing iron mineralization inside *So*Bfr12^WT^ and its variants were prepared by adding 1 mmol FeSO_4_ to lysates of cells grown to the mid‐exponential phase (OD_600_, 0.6–0.8), followed by incubation for 2 h at 25 °C. Native‐PAGE of the iron mineralized *So*Bfr12^WT^ and its variants was performed in a 6% (w v^−1^) nondenaturing polyacrylamide gel at 100 V for 1 h (at 4 °C). The gel was stained with a freshly prepared mixture of 2% K_4_Fe(CN)_6_ and 2% 11.6 mol HCl (1:1, v v^−1^) to check the iron‐incorporating ability of *So*Bfr12^WT^ and its variants by the Prussian blue assay.

### ICP‐MS Analysis

The Fe content in the samples was quantified using ICP‐MS (PerkinElmer NexlON 300XX) with an octupole reaction system (Yokogawa Analytical Systems). Pretreatment of the samples: Approximately 2 µmol protein samples were incubated overnight with 1 mmol FeSO₄ to facilitate iron loading. After incubation, the Fe‐loaded samples were subjected to size‐exclusion chromatography on a Superdex 200 Increase 10/30 column (GE Healthcare Life Sciences) in a low‐salt buffer (20 mmol Tris‐HCl, pH 8.0, 20 mmol NaCl) to remove excess iron and salts. The pooled protein samples, at a concentration of ≈1 mg mL^−1^, were diluted 1000‐fold with 2% HNO₃ and filtered through an aqueous filter membrane with a pore size of 0.22 µm in preparation for ICP‐MS analysis. The follow‐up test was completed by the Physical and Chemical Analysis Room of the Department of Agricultural Biological Environment, Zhejiang University.

### Kinetic Studies of Iron Oxidation and Release

To a protein solution of 1 µmol, 500 µmol (NH_4_)_2_Fe(SO_4_)_2_ freshly prepared in 0.015 mol HCl was added in 0.1 mol HEPES, pH 6.5. The iron oxidation was monitored by measuring the increase in the optical density at 310 nm, which specifically measures Fe^3+^ and the kinetic measurements were recorded from the time of the addition of iron to the protein.^[^ ^62^ ^]^ For iron release experiments, 1 µmol protein solution was mineralized with 500 µmol (NH_4_)_2_Fe(SO_4_)_2_ in 0.1 mol HEPES, pH 6.5, followed by incubation at 4 °C for 2 h. Iron release was initiated by the addition of 1 mmol ferrozine reagent prepared in 0.1 mol HEPES, pH 6.5, 250 mmol ascorbate. The quantity of released iron was measured kinetically by monitoring the absorbance of the Fe^2+^‐ferrozine complex at 570 nm.^[^ ^62^ ^]^

### Growth Competition Assays in Liquid Media

A single starter culture (S_T0_) was prepared for growth competition assays by mixing ≈1 × 10^7^ cells of each strain, grown independently to the stationary phase in MS medium. For the iron‐rich growth competition assay, an aliquot of S_T0_ was adjusted to 2 × 10^6^ cells mL^−1^ in a volume of 30 µL to inoculate 3 mL fresh medium irMS (containing 1 mmol FeSO_4_) and grown for 24 h until the stationary phase. For normal and iron‐limited competition experiments, 3 ml fresh medium MS and ilMS were used instead. After a round of incubation, 30 µL of the mixed culture was inoculated to fresh 3 mL of the same medium, and the rest was taken as the sample of S_T1_. In total, the procedure was repeated for 3 consecutive rounds. To determine the number of colonies, samples were serially diluted with fresh LB, and aliquots of 0.1 mL appropriately diluted samples were plated onto LB plates. After 1 day of growth, the colonies were counted according to their color distinction. Data was collected from four independent experiments.

### Phylogenetics Analysis

A protein similarity network was constructed using the EFI‐EST tool^[^ ^41^, ^42^ ^]^ with an alignment score of 50, and nodes were collapsed at a sequence identity of 65%. The network was visualized with the yFiles organic layout provided with the Cytoscape.^[^ ^63^ ^]^ For the phylogenetic analysis, proteins were selected representatively from the protein similarity network based on the presence of the InterPro domain IPR002024 (Bfr), IPR001519 (Ftn), IPR002177 (Dps), and IPR045236 (Rbr). The phylogenetic tree was performed using the phyML^[^ ^64^ ^]^ with MAFFT^[^ ^65^ ^]^ for the sequence alignment using the LG model. The conserved tree of ferritin‐like domain was constructed by MEGA X^[^ ^66^ ^]^ using the conserved sequences of Ftn, homo‐Bfr, hetero‐Bfr (DSD), and hetero‐Bfr (TD), with MAFFT for the sequence alignment and IQ with 1000 bootstrap replicates. Sequence alignments were achieved by MAFFT and visualized by Jalview.^[^ ^67^ ^]^

### Other Analyses

Most analyses were based on a minimum of four independent experiments, yielding biological replicates. The iron staining analyses were performed using three independent biological samples. Data were presented for either all replicates or presented as mean ± Standard Error of the Mean. Pairwise comparisons were conducted using Student's *t*‐test, with a *p*‐value below 0.05 considered statistically significant. Graphics and statistical analysis were performed using the Prism v9.5.1 software (GraphPad Software LLC, San Diego, CA, USA), completing the statistical test indicated in the text and figure legends.

## Conflict of Interest

The authors declare no conflict of interest.

## Author Contributions

H.G. conceived and designed the whole project. Y.L. and J.C. designed and performed the experiments. Y.L. performed genetic manipulation, purification, model building, refinement, and physiological experiments. Y.L. and W.W. contributed to the data collection. Y.L., J.C., W.W., and X.Z. performed image processing and reconstructions. Y.L. and J.C. contributed to the manuscript writing. Y.L., J.C., and H.G. wrote the manuscript with input from all authors.

## Supporting information



Supporting Information

Supporting cif

## Data Availability

Data and materials availability: The structure model data generated in this study have been deposited in the Protein Data Bank (PDB) under the accession codes 9IIG. The cryo‐EM density map data generated in this study have been deposited in the Electron Microscopy Data Bank (EMDB) under the accession codes EMD‐60594. Source data for the original figures are provided as a Source Data file.
