# Supplementary material for: Unveiling Structural Heterogeneity and Evolutionary Adaptations of Heteromultimeric Bacterioferritin Nanocages
Source: Adv Sci (Weinh). 2025 Apr 1;12(20):2409957. doi: 10.1002/advs.202409957 (PMC12120770; doi:10.1002/advs.202409957)
Supplement: Supplementary file 1 — Supporting Information [file ADVS-12-2409957-s002.docx]

Supporting Information

**Unveiling structural heterogeneity and evolutionary adaptations of heteromultimeric bacterioferritin nanocages**

*Yingxi Li, Weiwei Wang, Wei Wang, Xing Zhang, Jinghua Chen,* Haichun Gao,**

**Supporting Information contains:**

**Figure S1-S15**

**Table S1-S5**

**References**

**
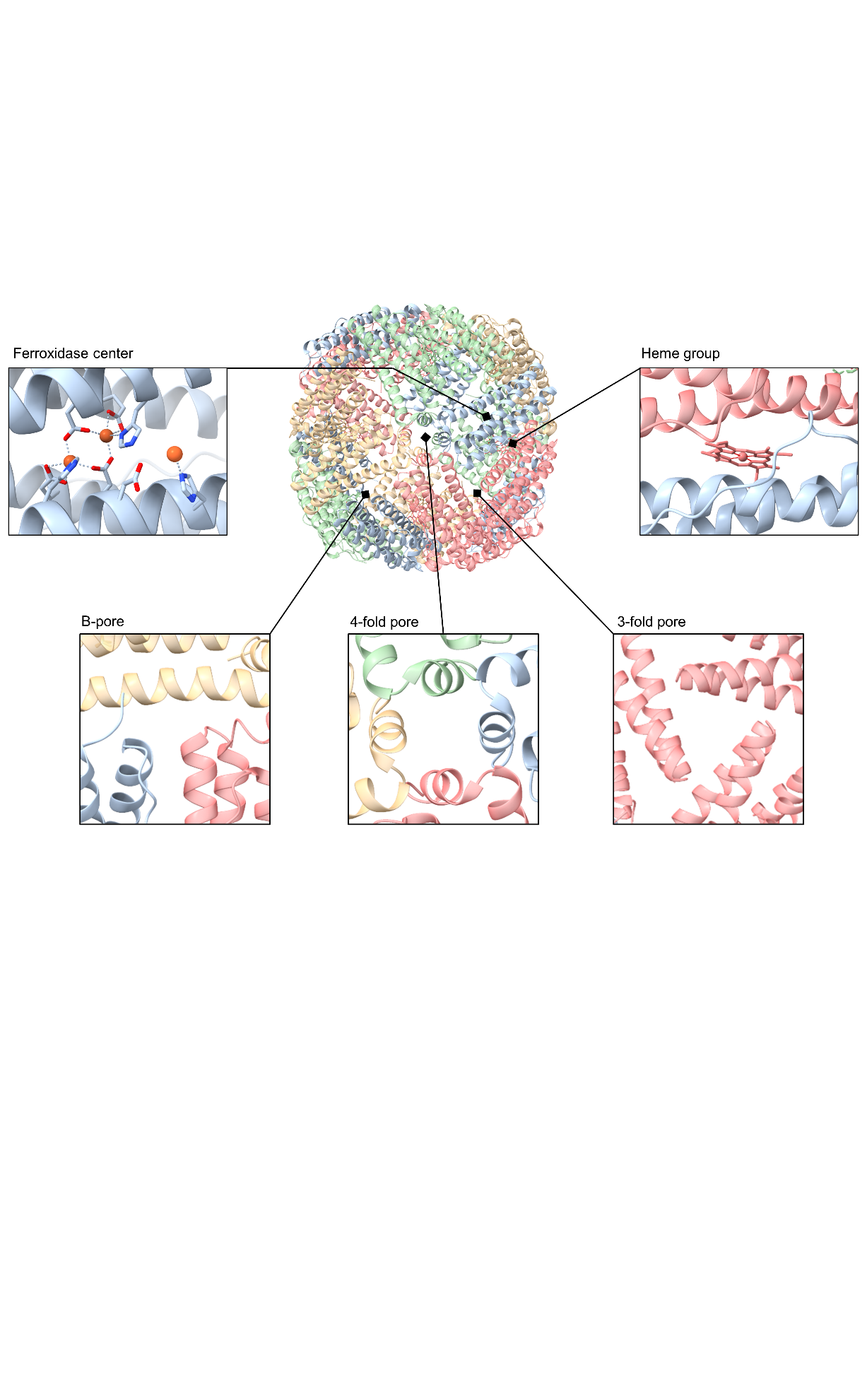
**

Figure S1. Overall view of homo-Bfr (*Pa*BfrB, PDB ID: 4TOH). Homo-Bfr is a nearly spherical molecule assembled from 24 identical subunits and 12 hemes. Each subunit harbors a FC, and each heme is at the interface of two subunits; iron in the FCs is shown as orange spheres. The interior cavity is in contact with the exterior via B-pores, 4-fold pores, and 3-fold pores.

**
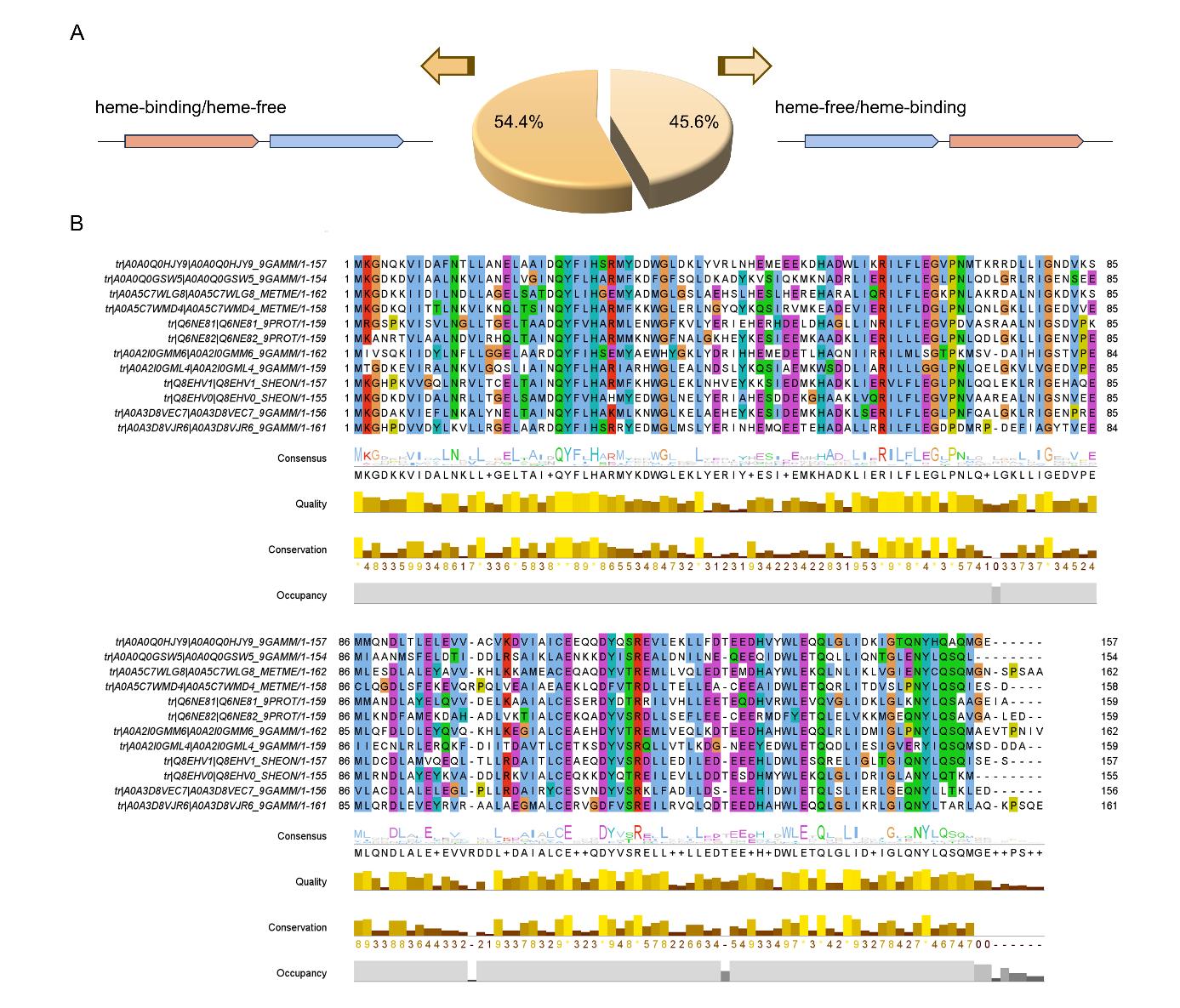
**

Figure S2. Two patterns of the TD-type *bfr* genes and multiple sequence alignment of hetero-Bfrs. A) Proportion of relative number of two patterns of the TD-type *bfr* genes. The initial 1,000 *bfr* genes in the Bfr cluster were obtained using the EFI-GNT tool,^[1, 2]^ and subsequently, 206 TD-type *bfr* genes were selected for statistical analysis. Among them, 112 *bfr* genes are in the order of “heme-binding/heme-free”, while 94 *bfr* genes are in the order of “heme-free/heme-binding”. The *bfr* genes encoding heme-binding and heme-free subunits are coloured in salmon and steel blue, respectively. B) Multiple sequence alignment of representative hetero-Bfrs from Fig. 1 is displayed on jalview.^[3]^ The clustal colour scheme is used for the ClustalW multiple sequence alignment.^[4]^ *Pseudoalteromonas* sp. P1-25 (Uniprot ID: A0A0Q0GSW5/A0A0Q0HJY9), *Psychrobacter* sp. 4Dc (A0A2I0GML4/A0A2I0GMM6), *Lysobacter soli* (A0A3D8VEC7/A0A3D8VJR6), *Methylophilus methylotrophus* (A0A5C7WLG8/A0A5C7WMD4), *Magnetospirillum gryphiswaldense* (Q6NE81/Q6NE82), *Shewanella oneidensis* (Q8EHV0/Q8EHV1).

**
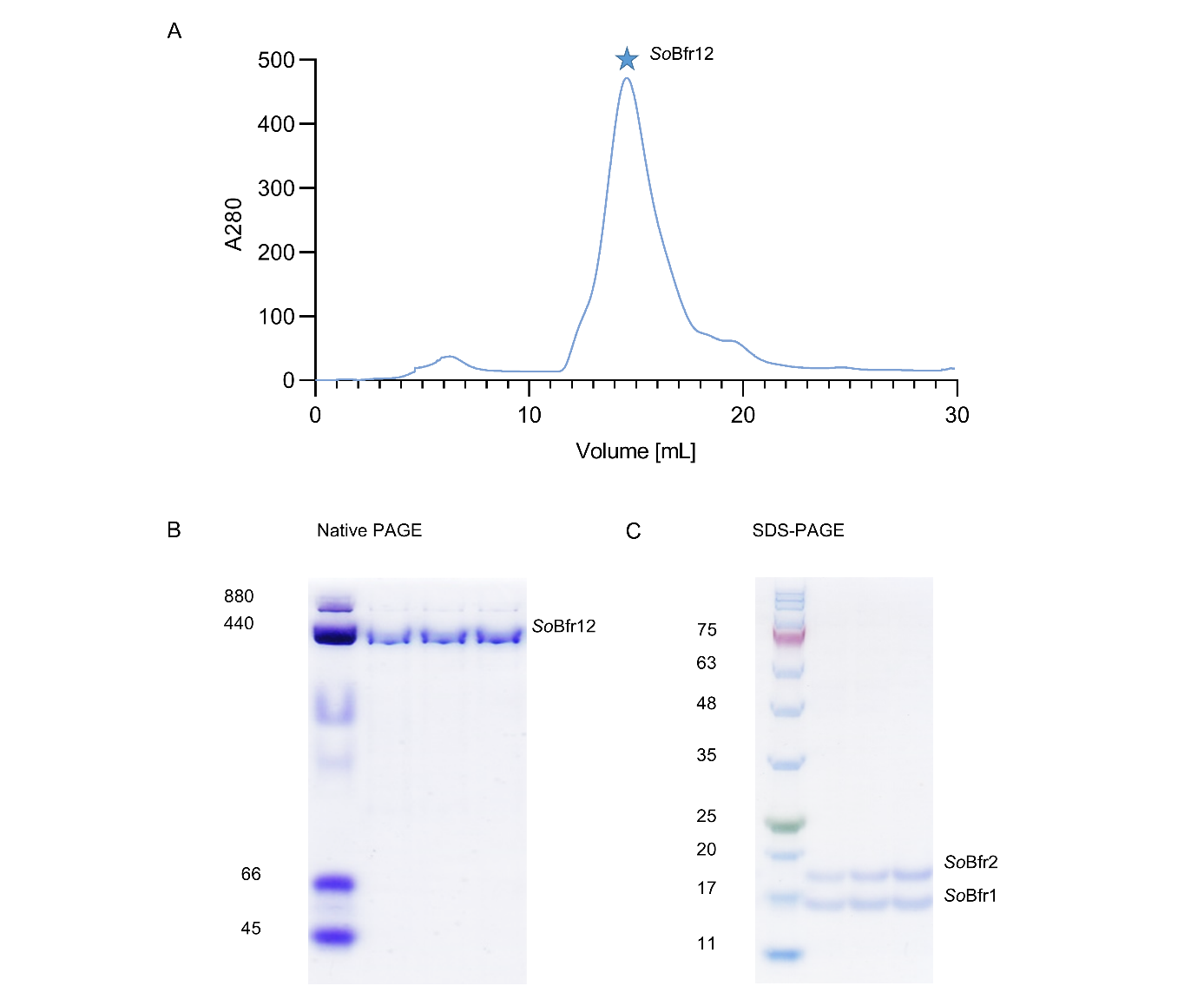
**

Figure S3. Sample preparation of *So*Bfr12 for cryo-EM. A) The chromatography profile of *So*Bfr12 size exclusion chromatography (SEC). B) the Native PAGE analysis of prepared *So*Bfr12 samples. c the SDS-PAGE analysis of prepared *So*Bfr12 samples.


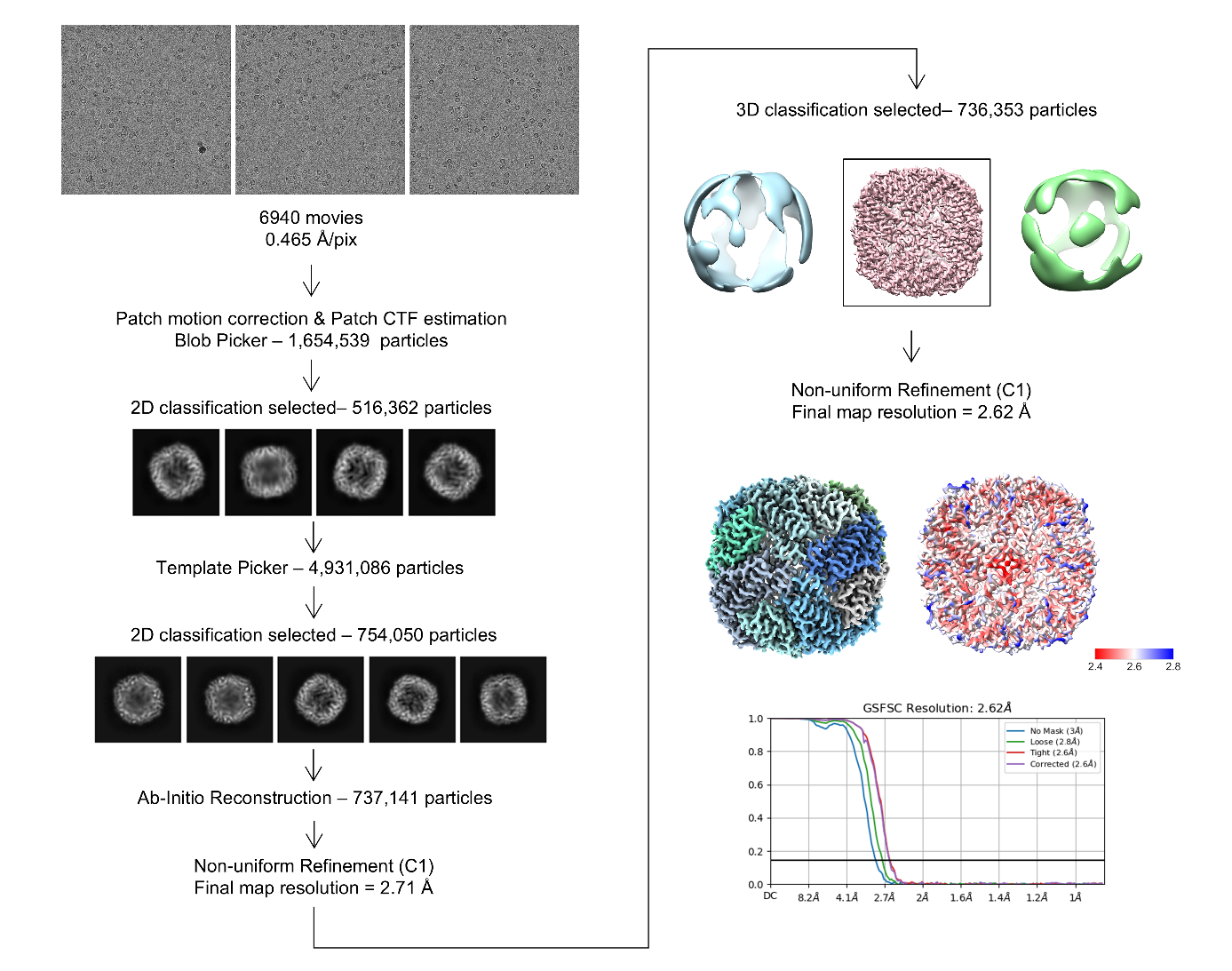


Figure S4. Cryo-EM data processing of *So*Bfr12. Fourier shell correlation (FSC) curve, showing the final resolution of 2.62 Å based on the golden standard FSC=0.143. Local resolution distribution of the density map is at bottom right.

**
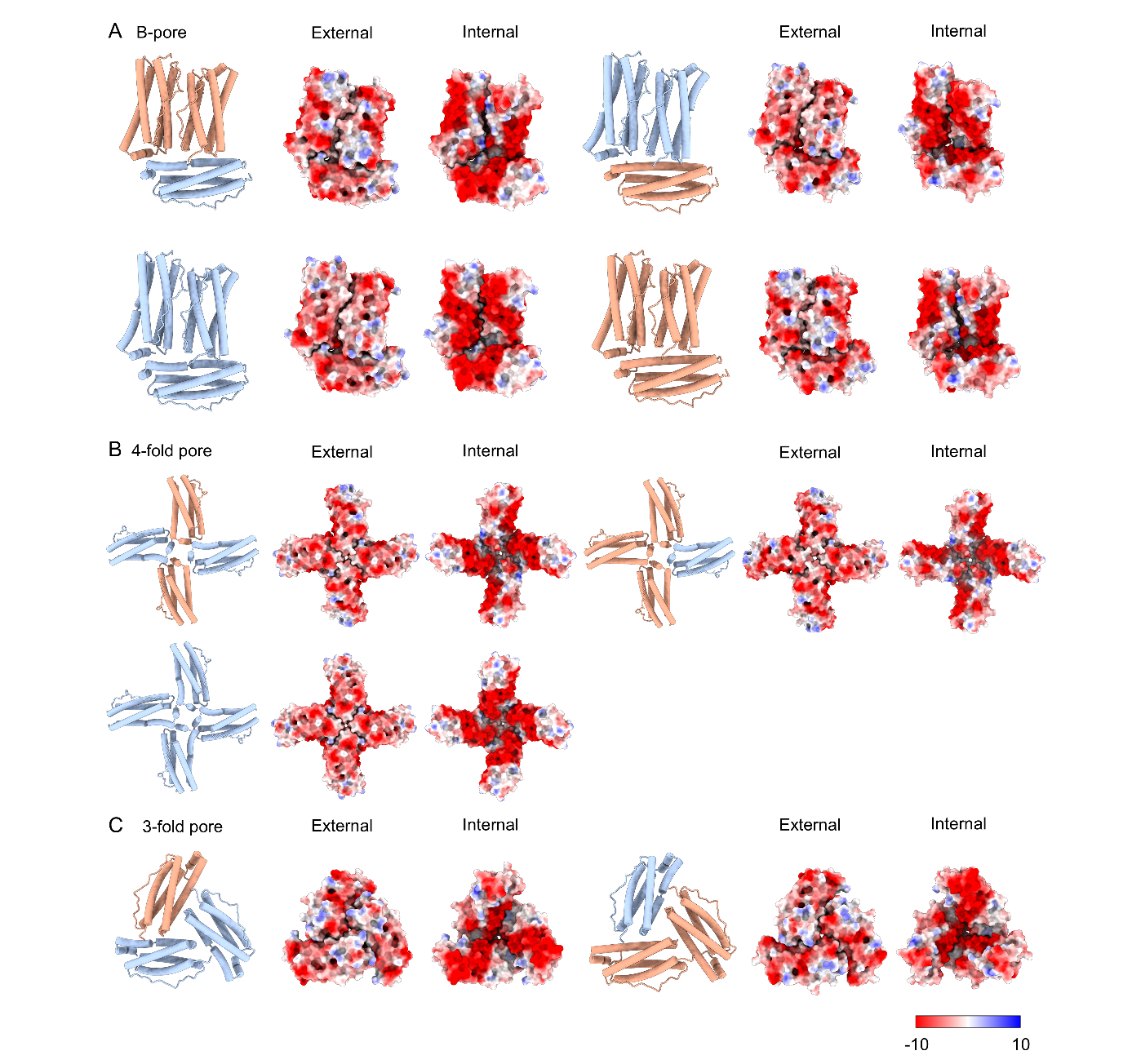
**

Figure S5. The external and internal surface electrostatic potential of the B-pores (A), 4-fold pores (B) and 3-fold pores (C) of *So*Bfr12. The corresponding structure, external and internal surface electrostatic potential of the subtype Ⅰ-Ⅳ B-pores, subtype Ⅰ-Ⅲ 4-fold pores and subtype Ⅰ-Ⅱ 3-fold pores are shown in order, respectively. *So*Bfr2 subunit is colored in salmon and *So*Bfr1 subunit is colored in steel blue.

**
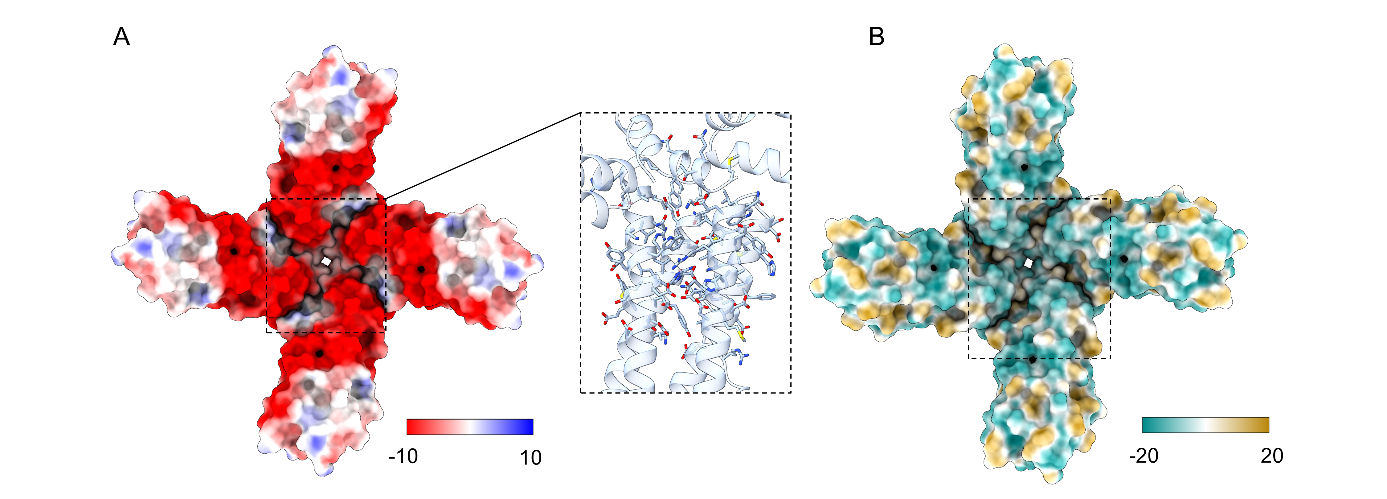
**

Figure S6. The surface electrostatic potential and hydrophobicity of the subtype Ⅰ 4-fold pore in *So*Bfr12. A) The surface electrostatic potential of the subtype Ⅰ 4-fold pore. The surface electrostatic potential is calculated by ChimeraX according to Coulomb's law.^[5]^ The close-in view shows the negatively charged and hydrophilic residues near the region of subtype Ⅰ 4-fold pore. B) The surface hydrophobicity of subtype Ⅰ 4-fold pore. *So*Bfr1 subunit is colored in steel blue.

**
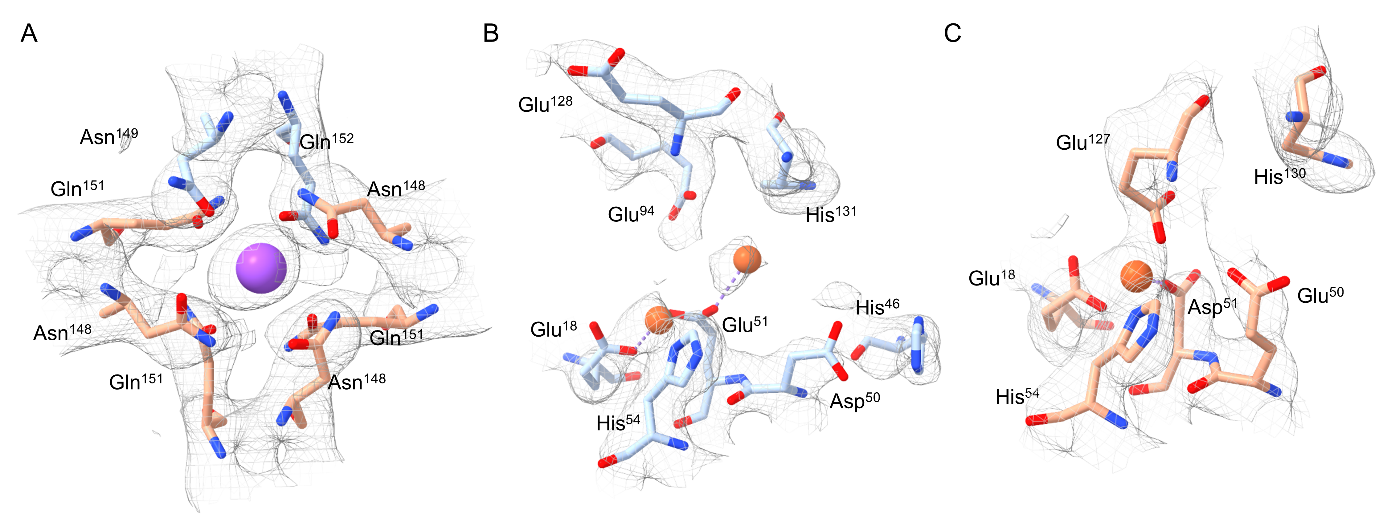
**

Figure S7. The cryo-EM density map of *So*Bfr12. A) The density map of *So*Bfr12 showing the Na ion in the center of the 4-fold pore and the coordinating residues Asn^148^, Gln^151^ from *So*Bfr2 and Asn^149^, Gln^152^ from *So*Bfr2. B-C) Modeled iron atoms are shown in the density map of *So*Bfr12. Due to the low intensity of the corresponding peaks in the cryo-EM map, these may represent potential iron-binding sites rather than confirmed iron atoms.

**
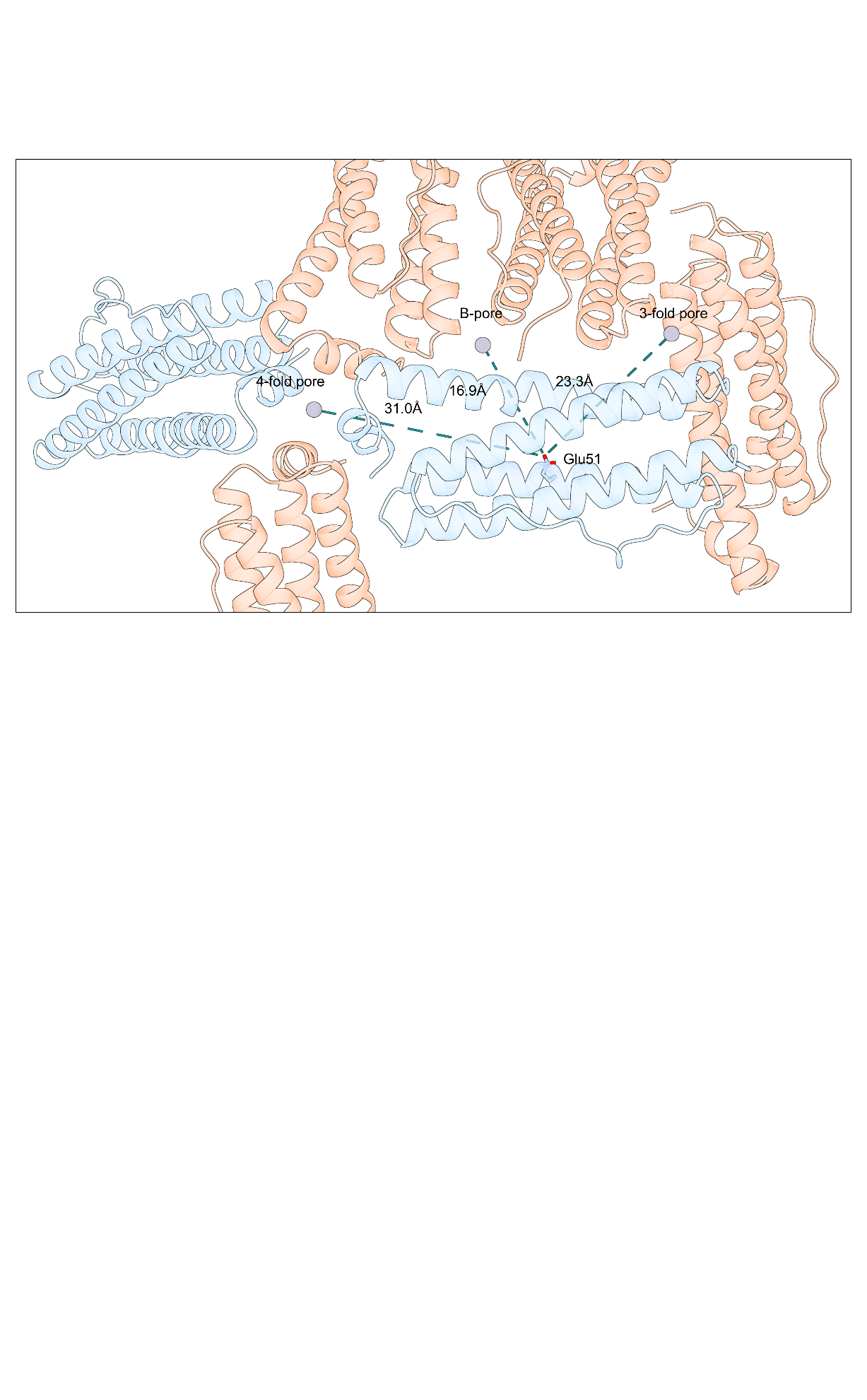
**

Figure S8. Distances from the FCs to the 4-fold, 3-fold and B-pores. The center of the pore is marked with a purple sphere. The distance (dark cyan) from the residues Glu^51^ in *So*Bfr1 FC to the B-pore is 16.9 Å, to the 4-fold pore is 31 Å, and to the 3-fold pore is 23.3 Å. *So*Bfr2 subunit is colored in salmon and *So*Bfr1 subunit is colored in steel blue.

**
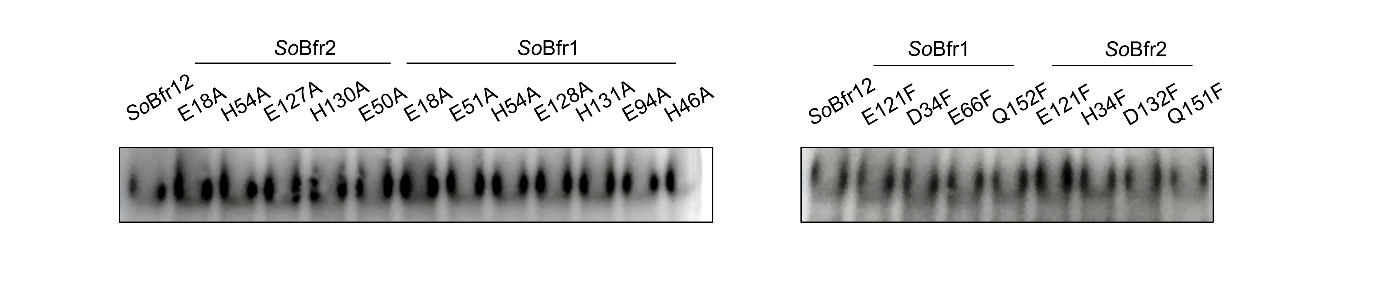
**

Figure S9. Native PAGE Western Blot analysis of *So*Bfr12 WT and relevant mutants.

**
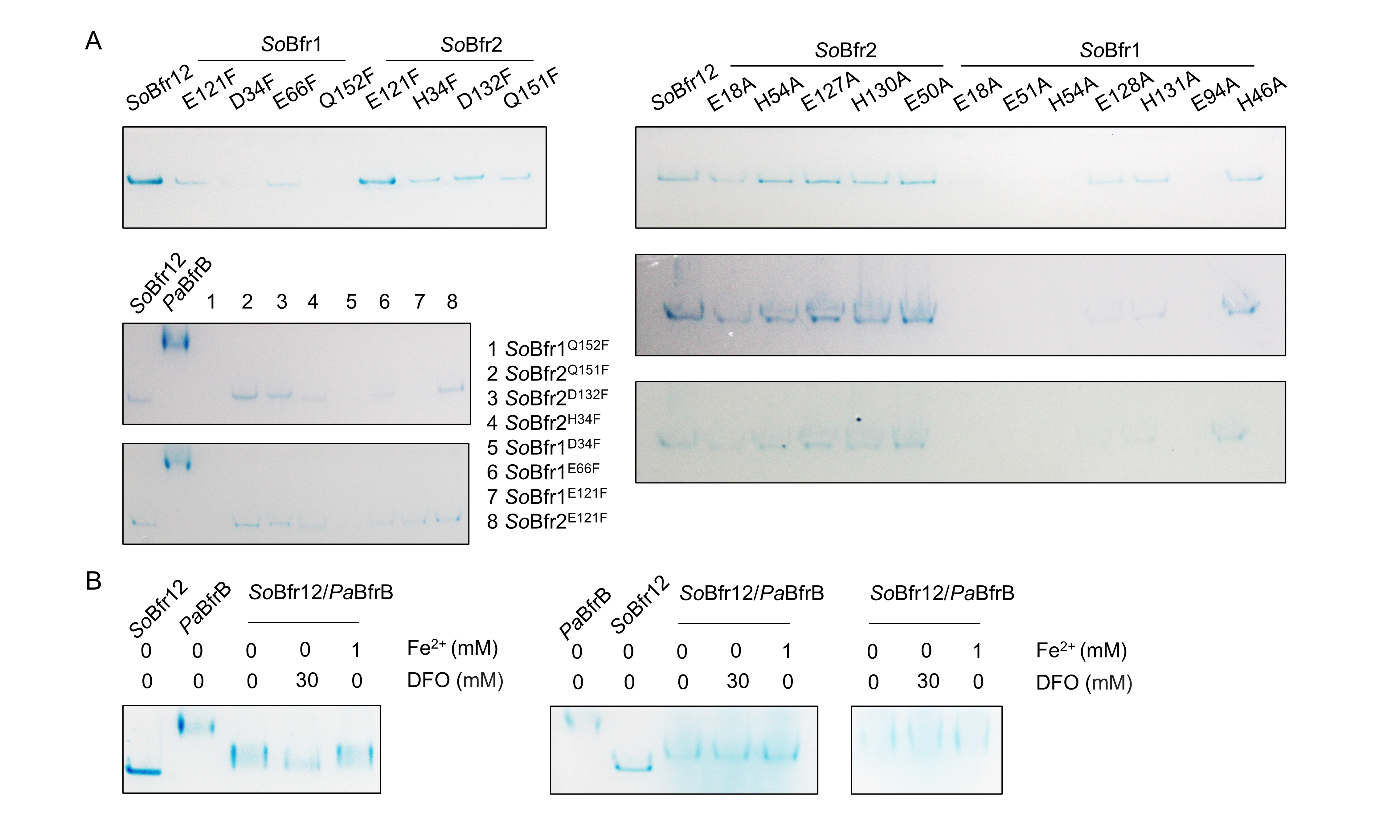
**

Figure S10. Iron staining results of *Pa*BfrB, *So*Bfr12/*Pa*BfrB, *So*Bfr12 WT and the relevant mutants. A）Iron staining results of *So*Bfr12 WT and the relevant mutants. B) Iron staining results of *So*Bfr12 WT, *Pa*BfrB and *So*Bfr12/*Pa*BfrB with different iron condition. The iron staining experiment was performed in three independent biological replicates for each condition.

**
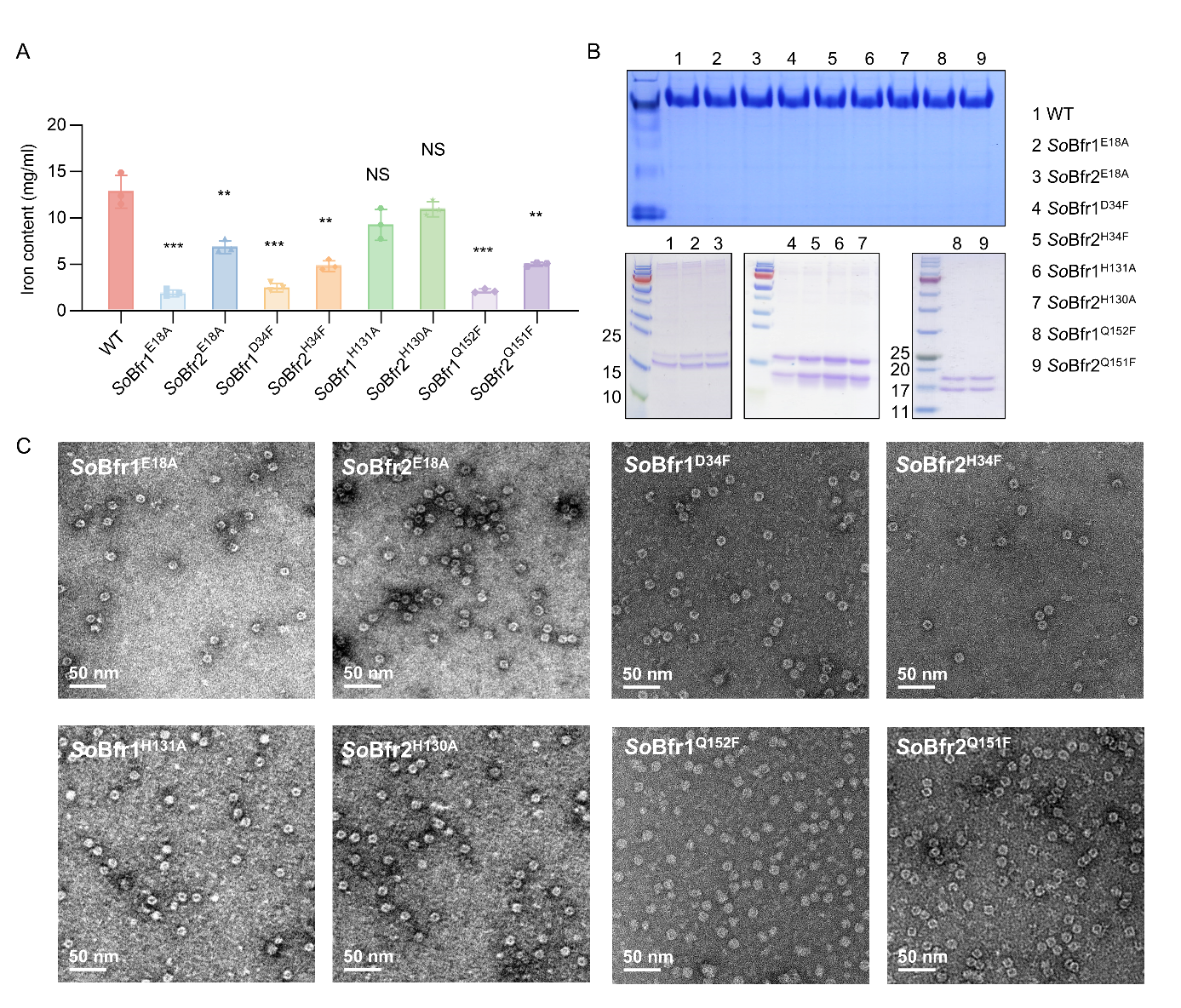
**

Figure S11. ICP-MS analysis of iron content and negative staining images of *So*Bfr12 WT and eight selected mutants. A) ICP-MS measurements of iron content for *So*Bfr12 WT and mutants, expressed as iron (Fe) concentration (mg ml^-1^) relative to the ~1 mg ml^-1^ protein concentration. Data are presented as the mean ± SD (n = 3 independent experiments). The significant difference was evaluated by two-tailed unpaired Student’s *t*-test, *, *p* < 0.05; **, *p* < 0.01; ***, *p* < 0.001. B) Top is the Native PAGE analysis, and bottom is the SDS-PAGE analysis for *So*Bfr12 WT and mutants. The significant difference was evaluated by Two-tailed unpaired Student’s t-test. C) Negative staining images of eight selected mutants.

**
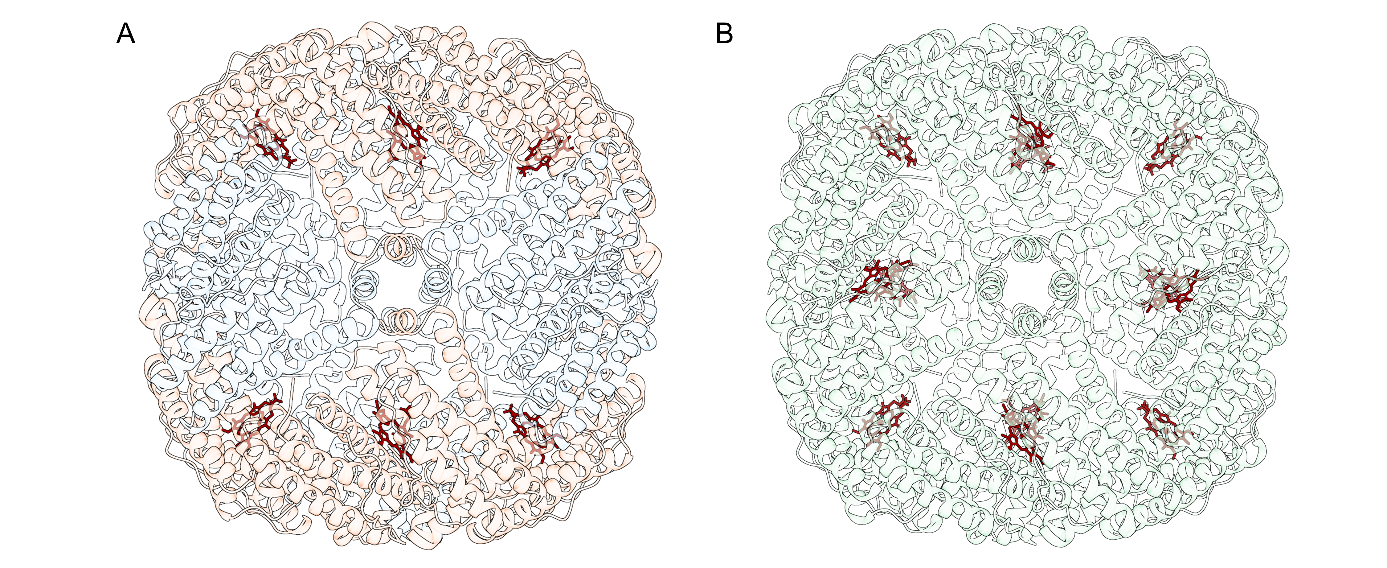
**

Figure S12. Distributions of heme groups in homo and hetero Bfrs. A) Structure of *So*Bfr12 and the distribution of the heme molecules. B) Structure of *Pa*BfrB (PDB ID: 4TOH) and the distribution of the heme molecules. *So*Bfr2 subunit is colored in salmon, *So*Bfr1 subunit is colored in steel blue, and *Pa*BfrB subunit is colored in sea green.

**
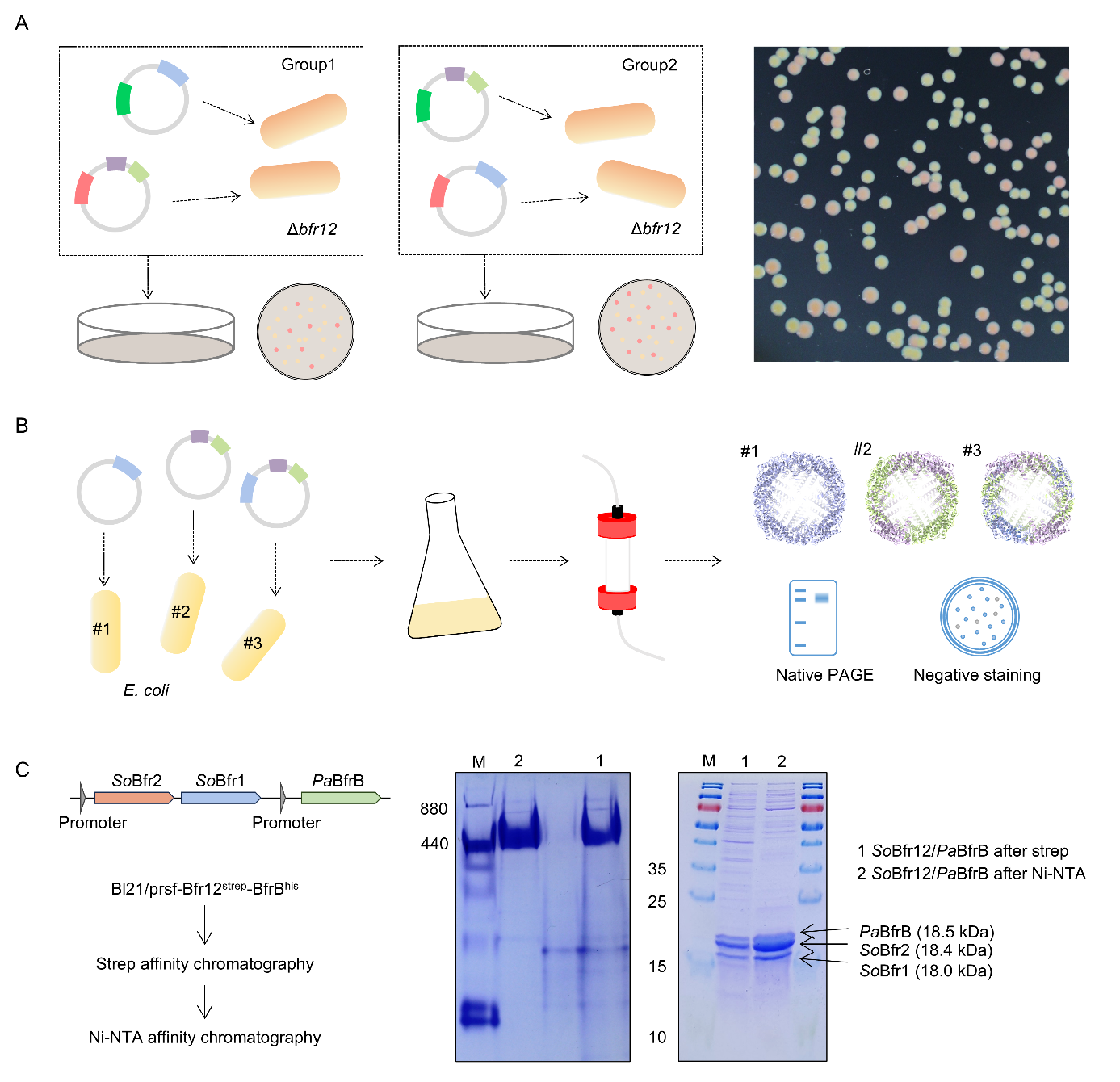
**

Figure S13. Flowchart of the functional experiment for *So*Bfr12 and *Pa*BfrB. A) Scheme of the growth competitiveness experiment. Group 1 consists of eGFP labeled Δ*bfr12*/p*PabfrB* and mScarlet labeled Δ*bfr12*/p*Sobfr12*; group 2 consists of eGFP labeled Δ*bfr12*/*pSobfr12* and mScarlet labeled Δ*bfr12*/p*PabfrB*. Diagram of colony distribution is shown on the right. mScarlet labeled strains with red color are easy to recognize on the plates. B) Scheme of the synthesis process of different types of Bfrs. #1 is the strain overexpressing the homo-Bfr, #2 is the strain overexpressing the hetero-Bfr, and #3 is the strain overexpressing the homo- and hetero-Bfr. Later, samples are checked by Native-PAGE and TEM. *bfr1* gene is colored in medium purple, *bfr2* gene is colored in light green, *bfrb* gene is colored in blue, eGFP coding gene is colored in green, and mScarlet coding gene is colored in red. C) Expression and purification of *So*Bfr12/*Pa*BfrB. (Left) a schematic showing the gene arrangement on the plasmid and purification procedure. Native PAGE (Medium) and SDS-PAGE (Right) analysis of *So*Bfr12/*Pa*BfrB after Strep and Ni-NTA affinity chromatography.

**
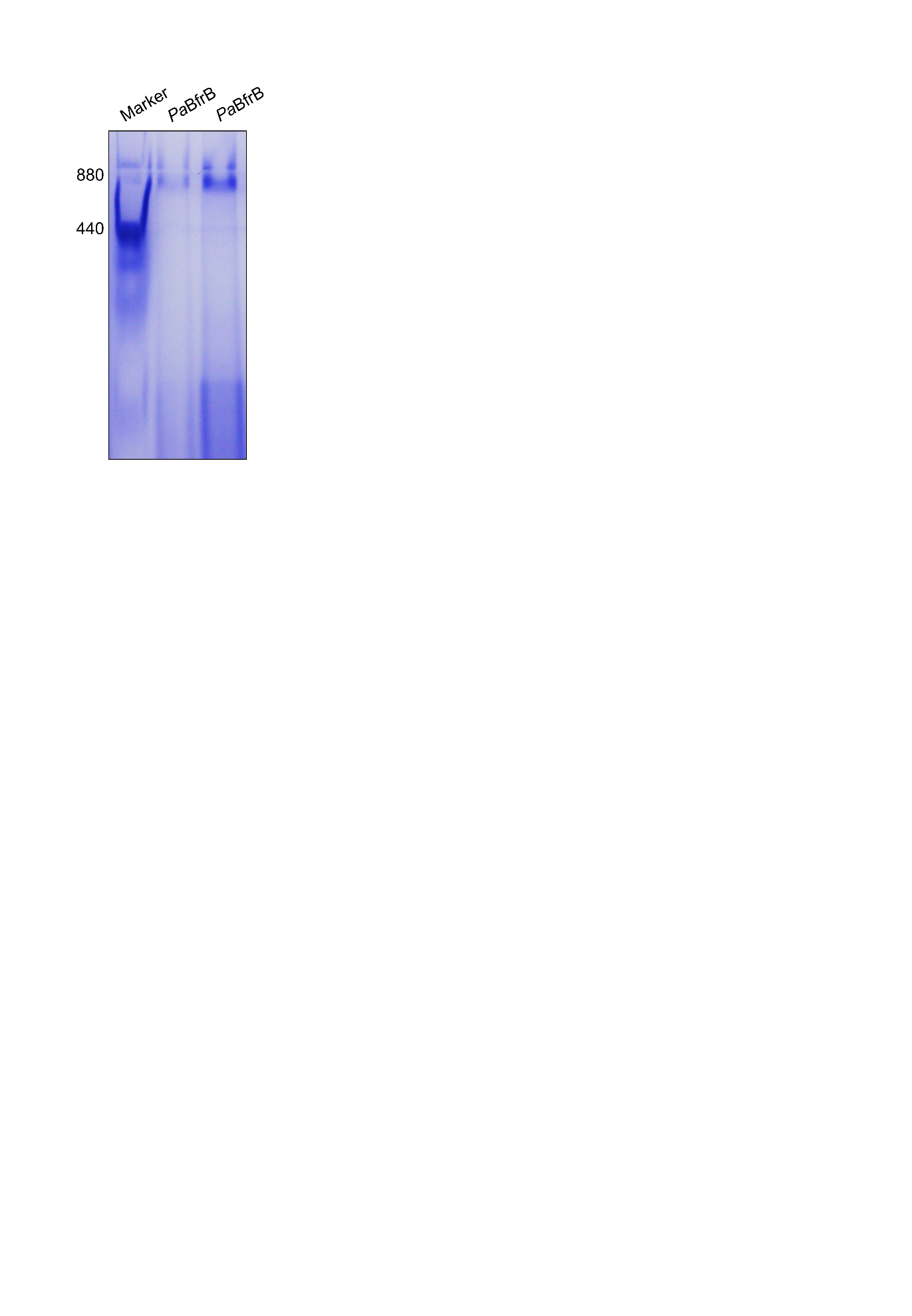
**

Figure S14. Native PAGE analysis of *Pa*BfrB after purification.

**
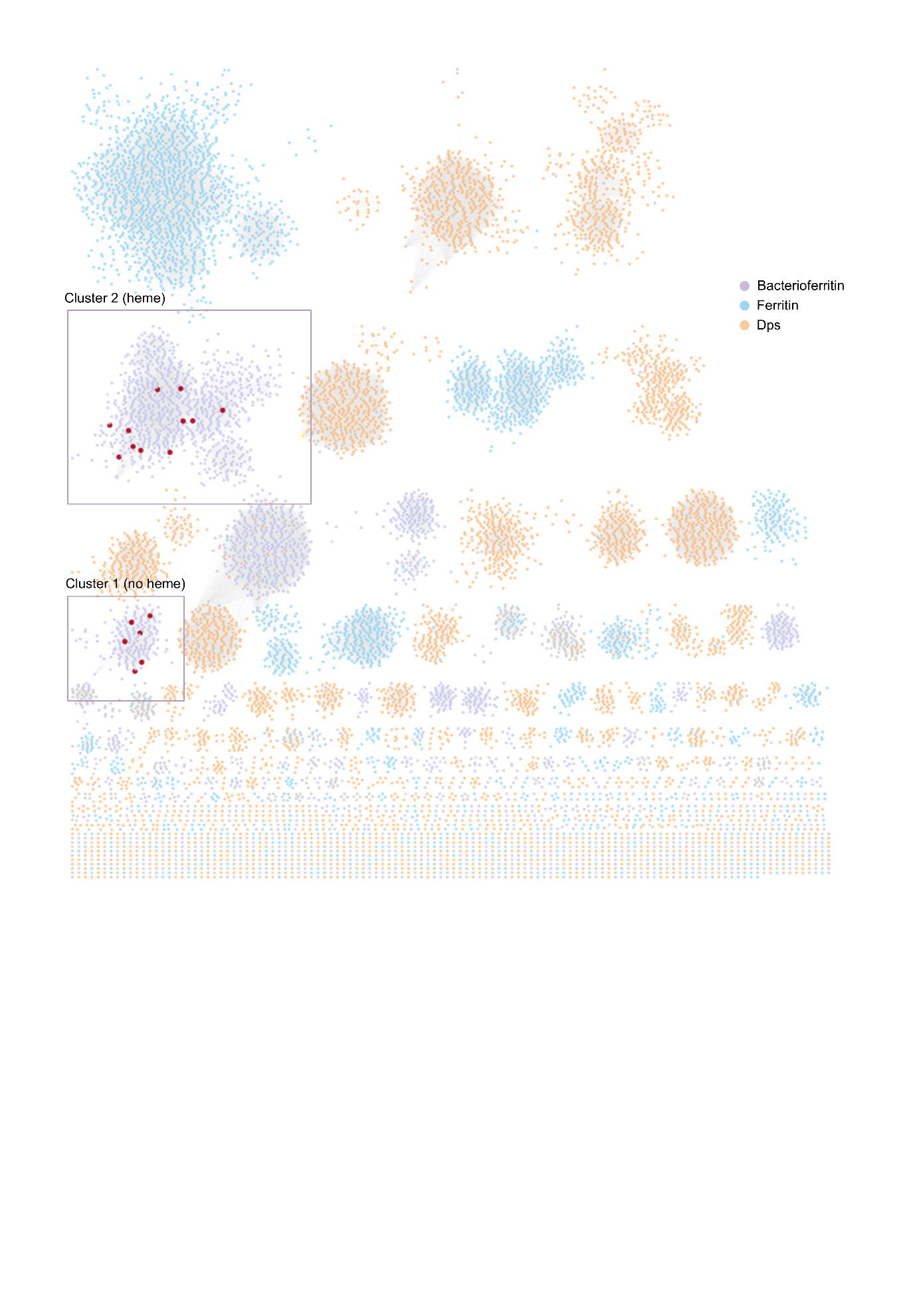
**

Figure S15. UniRef90 cluster SSN for ferritin superfamily in bacteria. The protein similarity network was constructed using the EFI-EST tool (https://efi.igb.illinois.edu/efi-est/)^[1, 2]^ with an alignment score of 50 and nodes were collapsed at a sequence identity of 65%. Detailed information about sequences involved in SSN is provided in a Source Data file.

Table S1. Cryo-EM data collection, refinement and validation statistics.

| **Data collection and processing** | |
| --- | --- |
| Magnification | 130,000 |
| Voltage (kV) | 300 |
| electron exposure (e^−^A^−2^) | 50 |
| exposure time (s) | 4.5 |
| defocus range (um) | -1 to -1.5 |
| pixel size (Å) | 0.465 |
| symmetry imposed | C1 |
| No. of images | 6,940 |
| No. of particles used | 736,353 |
| map resolution (Å) | 2.62 |
| FSC threshold | 0.143 |
| **Refinement** | |
| Model used | AlphaFold3 |
| Model composition |  |
| Non-hydrogen atoms | 30,870 |
| Protein residues | 3,744 |
| Chains | 24 |
| Ligands | HEM: 6, Na:6 |
| R.m.s. deviations (PHENIX) |  |
| Bond length (Å) | 0.008 |
| Bond angles (°) | 0.740 |
| **Validation** |  |
| MolProbity score | 1.49 |
| Clashscore | 9.25 |
| Rotamer outliers (%) | 0.00 |
| Ramachandran plot |  |
| Favored (%) | 99.35 |
| Allowed (%) | 0.65 |
| Disallowed (%) | 0.00 |

Table S2**.** Amino acid residues around B-, 4-fold, and 3-fold pores.

|  | Subtype | Interacting residues |
| --- | --- | --- |
| B-pores | Ⅰ | *So*Bfr1^E136^; *So*Bfr1^Y133^; *So*Bfr1^E66^; *So*Bfr1^D34^ |
|  | Ⅱ | *So*Bfr2^D132^; *So*Bfr2^E135^; *So*Bfr2^E66^; *So*Bfr2^H34^ |
|  | Ⅲ | *So*Bfr1^E136^; *So*Bfr1^Y133^; *So*Bfr2^E66^; *So*Bfr2^H34^ |
|  | Ⅳ | *So*Bfr2^D132^; *So*Bfr2^E135^; *So*Bfr1^E66^; *So*Bfr1^D34^ |
| 4-fold pores | Ⅰ | (*So*Bfr1^Q152^; *So*Bfr1^N149^)*4 |
|  | Ⅱ | *So*Bfr1^Q152^; *So*Bfr1^N149^; (*So*Bfr2^Q151^; *So*Bfr2^N148^)*3 |
|  | Ⅲ | (*So*Bfr2^Q151^; *So*Bfr2^N148^)*2; (*So*Bfr1^Q152^; *So*Bfr1^N149^)*2 |
| 3-fold pores | Ⅰ | *So*Bfr2^E118^; *So*Bfr2^E121^; (*So*Bfr1^E118^; *So*Bfr01^E121^)*2 |
|  | Ⅱ | *So*Bfr1^E118^; *So*Bfr1^E121^; (*So*Bfr2^E118^; *So*Bfr2^E121^)*2 |

Table S3. Amino acid residues (contributing to the electrostatics and ferroxidase activity in *So*Bfr12) chosen for site directed mutagenesis.

| ***So*Bfr1** | **Residues selected** |
| --- | --- |
| 4-fold pore | Q151 |
| 3-fold pore | E121 |
| B-pore | D34, E66 |
| IS iron binding site | H46 |
| ***So*Bfr2** | **Residues selected** |
| 4-fold pore | Q152 |
| 3-fold pore | E121 |
| B-pore | H34, D132 |
| Ferroxidase center | E18, H54, E127, H130, E50 |
| IS iron binding site | E50 |
| Heme binding site | M52 |

Table S4. Strains and plasmids used in this study.

| **Strains or plasmids** | **Description** | **Source/reference** |
| --- | --- | --- |
| Strains |  |  |
| *E. coli* |  |  |
| BL21 | Recombinant protein expression host strain | Novagen |
| DH5α | Host strain for plasmids | Lab stock |
| WM3064 | Donor strain for conjugation: ΔdapA | W. Metcalf, UIUC |
| *S. oneidensis* |  |  |
| MR-1 | WT | Lab stock |
| HG1111-1112 | Δ*bfr12* derived from MR-1 | ^[6]^ |
| *P. aeruginosa* |  |  |
| PAO1 | WT | Lab stock |
| Plasmids |  |  |
| pET-28a(+) | Km^r^, recombinant protein expression vector | Novagen |
| pET-28a(+)-Bfr12^strep^ | Inducible expression of *So*Bfr12^strep^ | This study |
| pET-28a(+)-BfrB^his^ | Inducible expression of *Pa*BfrB^his^ | This study |
| pRSFDuet-1 | Km^r^, IPTG-inducible Ptac expression vector | Novagen |
| pRSFDuet-1-Bfr12^strep^-BfrB^his^ | Inducible expression of *So*Bfr12^strep^/BfrB^his^ | This study |
| pHGEN-Ptac^Km^ | Km^r^, IPTG-inducible Ptac expression vector | ^[7]^ |
| pHGEN-Ptac^Km^-Bfr12 | Inducible expression of *So*Bfr12 | This study |
| pHGEN-Ptac^Km^-BfrB | Inducible expression of *Pa*BfrB | This study |
| pHGEN-Ptac^Km^-Bfr12^strep^ | Inducible expression of *So*Bfr12^strep^ | This study |
| pHGEN-Ptac^Km^-Bfr12^strep_Bfr1 E121F^ | Inducible expression of *So*Bfr12^strep_Bfr1 E121F^ | This study |
| pHGEN-Ptac^Km^-Bfr12^strep_Bfr1 D34F^ | Inducible expression of *So*Bfr12^strep_Bfr1 D34F^ | This study |
| pHGEN-Ptac^Km^-Bfr12^strep_Bfr1 E66F^ | Inducible expression of *So*Bfr12^strep_Bfr1 E66F^ | This study |
| pHGEN-Ptac^Km^-Bfr12^strep_Bfr1 Q152F^ | Inducible expression of *So*Bfr12^strep_Bfr1 Q152F^ | This study |
| pHGEN-Ptac^Km^-Bfr12^strep_Bfr1 E18A^ | Inducible expression of *So*Bfr12^strep_Bfr1 E18A^ | This study |
| pHGEN-Ptac^Km^-Bfr12^strep_Bfr1 E51A^ | Inducible expression of *So*Bfr12^strep_Bfr1 E51A^ | This study |
| pHGEN-Ptac^Km^-Bfr12^strep_Bfr1 H54A^ | Inducible expression of *So*Bfr12^strep_Bfr1 H54A^ | This study |
| pHGEN-Ptac^Km^-Bfr12^strep_Bfr1 E128A^ | Inducible expression of *So*Bfr12^strep_Bfr1 E128A^ | This study |
| pHGEN-Ptac^Km^-Bfr12^strep_Bfr1 H131A^ | Inducible expression of *So*Bfr12^strep_Bfr1 H131A^ | This study |
| pHGEN-Ptac^Km^-Bfr12^strep_Bfr1 E94A^ | Inducible expression of *So*Bfr12^strep_Bfr1 E94A^ | This study |
| pHGEN-Ptac^Km^-Bfr12^strep_Bfr1 H46A^ | Inducible expression of *So*Bfr12^strep_Bfr1 H46A^ | This study |
| pHGEN-Ptac^Km^-Bfr12^strep_Bfr2 E121F^ | Inducible expression of *So*Bfr12^strep_Bfr2 E121F^ | This study |
| pHGEN-Ptac^Km^-Bfr12^strep_Bfr2 H34F^ | Inducible expression of *So*Bfr12^strep_Bfr2 H34F^ | This study |
| pHGEN-Ptac^Km^-Bfr12^strep_Bfr2 D132F^ | Inducible expression of *So*Bfr12^strep_Bfr2 D132F^ | This study |
| pHGEN-Ptac^Km^-Bfr12^strep_Bfr2 Q151F^ | Inducible expression of *So*Bfr12^strep_Bfr2 Q151F^ | This study |
| pHGEN-Ptac^Km^-Bfr12^strep_Bfr2 E18A^ | Inducible expression of *So*Bfr12^strep_Bfr2 E18A^ | This study |
| pHGEN-Ptac^Km^-Bfr12^strep_Bfr2 H54A^ | Inducible expression of *So*Bfr12^strep_Bfr2 H54A^ | This study |
| pHGEN-Ptac^Km^-Bfr12^strep_Bfr2 E127A^ | Inducible expression of *So*Bfr12^strep_Bfr2 E127A^ | This study |
| pHGEN-Ptac^Km^-Bfr12^strep_Bfr2 H130A^ | Inducible expression of *So*Bfr12^strep_Bfr2 H130A^ | This study |
| pHGEN-Ptac^Km^-Bfr12^strep_Bfr2 E50A^ | Inducible expression of *So*Bfr12^strep_Bfr2 E50A^ | This study |
| pHGEN-Ptac^Km^-Bfr12^strep_Bfr2 M52L^ | Inducible expression of *So*Bfr12^strep_Bfr2 M52L^ | This study |
| pHGEN-Ptac^Gm^ | Km^r^, IPTG-inducible Ptac expression vector | Lab stock |
| pHGEN-PtacG^m^-mScarlet | Inducible expression of mScarlet | Lab stock |

Table S5. Primers used in this study.

| **Primers** | | **Sequence** |
| --- | --- | --- |
| pET-28a(+)-Bfr12^strep^-F | CCGGAATTCCACTCATTTCAAGGATCGTA | |
| pET-28a(+)-Bfr12^strep^-R | CGCGGATCCTTATTTTTCGAACTGCGGGTGGCTCCACATCTTTGTTTGCAGATAATTCG | |
| pET-28a(+)-BfrB^strep^-F | CATGCCATGGCGATGAAAGGCGACAAGAAAGT | |
| pET-28a(+)-BfrB^strep^-F | CGCGGATCCTCATTTTTCGAACTGCGGGTGGCTCCAGTCGTCTTCGTGCATGTGCG | |
| pRSFDuet-1-Bfr12^strep^-BfrB^his^-12F | CATGCCATGGCGAAAGGTCATCCAAAAGTG | |
| pRSFDuet-1-Bfr12^strep^-BfrB^his^-12R | CCCAAGCTTTTATTTTTCGAACTGCGGGTGGCTCCACATCTTTGTTTGCAGATAATTCG | |
| pRSFDuet-1-Bfr12^strep^-BfrB^his^-BF | GGAATTCCATATGGCGAAAGGCGACAAGAAAGTCAT | |
| pRSFDuet-1-Bfr12^strep^-BfrB^his^-BR | CCGCTCGAGTCAGTGGTGATGATGGTGATGGTCGTCTTCGTGCATGTGCG | |
| pHGEN-Ptac^Km^-Bfr12-F | CCGGAGCTCGGCTGAAAGCTTGATTTAGA | |
| pHGEN-Ptac^Km^-Bfr12-R | CGCGGATCCAAAATGGCTGGCCTTGGTAG | |
| pHGEN-Ptac^Km^-BfrB-F | CCGGAGCTCCGATGAAAGGCGACAAGAAAGT | |
| pHGEN-Ptac^Km^-BfrB-R | CGCGGATCCTCAGTCGTCTTCGTGCATGTGCG | |
| pHGEN-Ptac^Km^-Bfr12^strep^-F | CCGGAATTCCACTCATTTCAAGGATCGTA | |
| pHGEN-Ptac^Km^-Bfr12^strep^-R | CGCGGATCCTTATTTTTCGAACTGCGGGTGGCTCCACATCTTTGTTTGCAGATAATTCG | |
| pHGEN-Ptac^Km^-Bfr12^strep_Bfr1 E121F^-F | ATCAAACCCGTGAGATCTTATTCGTCCTACTCGACGATACTGA | |
| pHGEN-Ptac^Km^-Bfr12^strep_Bfr1 E121F^-R | AATCTTTTTTCTGCTCACAG | |
| pHGEN-Ptac^Km^-Bfr12^strep_Bfr1 D34F^-F | TGCATGCCCATATGTACGAATTTTGGGGACTTAACGAACTTTA | |
| pHGEN-Ptac^Km^-Bfr12^strep_Bfr1 D34F^-R | CAAAATACTGATCCATAGCC | |
| pHGEN-Ptac^Km^-Bfr12^strep_Bfr1 E66F^-F | TGCAGCGTATTTTATTCCTCTTCGGTGTGCCCAATGTTGCGGC | |
| pHGEN-Ptac^Km^-Bfr12^strep_Bfr1 E66F^-R | CCAGTTTTGCCGCATGGCCT | |
| pHGEN-Ptac^Km^-Bfr12^strep_Bfr1 Q152F^-F | TTGGTTTAGCGAATTATCTGTTCACAAAGATGTGGAGCCACCC | |
| pHGEN-Ptac^Km^-Bfr12^strep_Bfr1 Q152F^-R | TGCGGTCGATTAAACCGAGC | |
| pHGEN-Ptac^Km^-Bfr12^strep_Bfr1 E18A^-F | TAAATCGACTCCTAACCGGGGCGTTATCGGCTATGGATCAGTA | |
| pHGEN-Ptac^Km^-Bfr12^strep_Bfr1 E18A^-R | ACGCATCGATGACGTCTTTA | |
| pHGEN-Ptac^Km^-Bfr12^strep_Bfr1 E51A^-F | TTGCCCACGAGTCAGATGACGCGAAAGGCCATGCGGCAAAACT | |
| pHGEN-Ptac^Km^-Bfr12^strep_Bfr1 E51A^-R | TGCGTTCATAAAGTTCGTTA | |
| pHGEN-Ptac^Km^-Bfr12^strep_Bfr1 H54A^-F | AGTCAGATGACGAGAAAGGCGCTGCGGCAAAACTGGTGC | |
| pHGEN-Ptac^Km^-Bfr12^strep_Bfr1 H54A^-R | CGTGGGCAATGCGTTCATAA | |
| pHGEN-Ptac^Km^-Bfr12^strep_Bfr1 E128A^-F | AAGTCCTACTCGACGATACTGCGTCTGACCATATGTATTGGCT | |
| pHGEN-Ptac^Km^-Bfr12^strep_Bfr1 E128A^-R | CTAAGATCTCACGGGTTTGA | |
| pHGEN-Ptac^Km^-Bfr12^strep_Bfr1 H131A^-F | TCGACGATACTGAGTCTGACGCTATGTATTGGCTTGAGAAGCA | |
| pHGEN-Ptac^Km^-Bfr12^strep_Bfr1 H131A^-R | GTAGGACTTCTAAGATCTCA | |
| pHGEN-Ptac^Km^-Bfr12^strep_Bfr1 E94A^-F | TGCGTAACGATCTCGCCTATGCATATAAAGTTGCCGATGATTT | |
| pHGEN-Ptac^Km^-Bfr12^strep_Bfr1 E94A^-R | GCATCTCTTCGACATTTGAA | |
| pHGEN-Ptac^Km^-Bfr12^strep_Bfr1 H46A^-F | AACTTTATGAACGCATTGCCGCCGAGTCAGATGACGAGAAAGG | |
| pHGEN-Ptac^Km^-Bfr12^strep_Bfr1 H46A^-R | CGTTAAGTCCCCAATCTTCG | |
| pHGEN-Ptac^Km^-Bfr12^strep_Bfr2 E121F^-F | ATGTCAGTCGTGATCTGCTGTTCGACATCCTTGAAGATGAAGA | |
| pHGEN-Ptac^Km^-Bfr12^strep_Bfr2 E121F^-R | AATCCTGCTCGGCTTCACAT | |
| pHGEN-Ptac^Km^-Bfr12^strep_Bfr2 H34F^-F | TCCACGCCAGAATGTTTAAGTTTTGGGGTCTTGAAAAGCTTAA | |
| pHGEN-Ptac^Km^-Bfr12^strep_Bfr2 H34F^-R | GAAAATATTGGTTAATGGCCG | |
| pHGEN-Ptac^Km^-Bfr12^strep_Bfr2 D132F^-F | AAGATGAAGAAGAGCATTTATTTTGGCTCGAGTCACAGCGGGA | |
| pHGEN-Ptac^Km^-Bfr12^strep_Bfr2 D132F^-R | CAAGGATGTCTTCCAGCAGA | |
| pHGEN-Ptac^Km^-Bfr12^strep_Bfr2 Q151F^-F | CCGGCATTCAAAACTACCTATTCTCGCAAATTAGTGAGTCATA | |
| pHGEN-Ptac^Km^-Bfr12^strep_Bfr2 Q151F^-R | TTAAGCCAATCAGCTCCCGC | |
| pHGEN-Ptac^Km^-Bfr12^strep_Bfr2 E18A^-F | TCAATCGGGTGCTTACCTGTGCGTTAACGGCCATTAACCAATA | |
| pHGEN-Ptac^Km^-Bfr12^strep_Bfr2 E18A^-R | GTTGTCCCACCACTTTTGGA | |
| pHGEN-Ptac^Km^-Bfr12^strep_Bfr2 H54A^-F | ATCGAAGATATGAAGGCTGCCGATAAGCTTATCGAGCGAGTGT | |
| pHGEN-Ptac^Km^-Bfr12^strep_Bfr2 H54A^-R | GGATTTTTTATATTCGACGTG | |
| pHGEN-Ptac^Km^-Bfr12^strep_Bfr2 E127A^-F | TGGAAGACATCCTTGAAGATGCAGAAGAGCATTTAGATTGGCT | |
| pHGEN-Ptac^Km^-Bfr12^strep_Bfr2 E127A^-R | GCAGATCACGACTGACATAA | |
| pHGEN-Ptac^Km^-Bfr12^strep_Bfr2 H130A^-F | TCCTTGAAGATGAAGAAGAGGCTTTAGATTGGCTCGAGTCACA | |
| pHGEN-Ptac^Km^-Bfr12^strep_Bfr2 H130A^-R | TGTCTTCCAGCAGATCACGA | |
| pHGEN-Ptac^Km^-Bfr12^strep_Bfr2 E50A^-F | TCGAATATAAAAAATCCATCGCAGATATGAAGCATGCCGATAA | |
| pHGEN-Ptac^Km^-Bfr12^strep_Bfr2 E50A^-R | CGTGATTAAGCTTTTCAAGACC | |
| pHGEN-Ptac^Km^-Bfr12^strep_Bfr2 M52L^-F | CGAGCGAGTGTTGTTTTTGG | |
| pHGEN-Ptac^Km^-Bfr12^strep_Bfr2 M52L^-R | ATAAGCTTATCGGCATGCTTCAGATCTTCGATGGATTTTTTAT | |

References

1. N. Oberg, R. Zallot, J. A. Gerlt, *J. Mol. Biol.* **2023**, 435, 168018.

2. R. Zallot, N. Oberg, J. A. Gerlt, *Biochemistry* **2019**, 58, 4169–4182.

3. A. M. Waterhouse, J. B. Procter, D. M. A. Martin, M. Clamp, G. J. Barton, *Bioinformatics* **2009**, 25, 1189–1191.

4. M. A. Larkin, G. Blackshields, N. P. Brown, R. Chenna, P. A. McGettigan, H. McWilliam, F. Valentin, I. M. Wallace, A. Wilm, R. Lopez, J. D. Thompson, T. J. Gibson, D. G. Higgins, *Bioinformatics* **2007**, 23, 2947–2948.

5. E. C. Meng, T. D. Goddard, E. F. Pettersen, G. S. Couch, Z. J. Pearson, J. H. Morris, T. E. Ferrin, *Protein Science* **2023**, 32, e4792.

6. H. Fu, L. Liu, Z. Dong, S. Guo, H. Gao, *Appl. Environ. Microbiol.* **2018**, 84, e00039-18.

7. N. T. Huy, D. T. Xuan Trang, D. T. Uyen, M. Sasai, S. Harada, K. Kamei, *Anal. Biochem.* **2005**, 344, 289–291.
